# Integration of Biomarker Polygenic Risk Score Improves Prediction of Coronary Heart Disease

**DOI:** 10.1016/j.jacbts.2023.07.006

**Published:** 2023-10-04

**Authors:** Jake Lin, Nina Mars, Yu Fu, Pietari Ripatti, Tuomo Kiiskinen, FinnGen study, Taru Tukiainen, Samuli Ripatti, Matti Pirinen

**Affiliations:** a Institute for Molecular Medicine Finland, Helsinki Institute of Life Science, University of Helsinki, Helsinki, Finland; b Health Sciences, Faculty of Social Sciences, Tampere University, Tampere, Finland; c Department of Public Health, University of Helsinki, Helsinki, Finland; d Broad Institute of Massachusetts Institute of Technology, Harvard University, Cambridge, Massachusetts, USA; e Massachusetts General Hospital, Cambridge, Massachusetts, USA; f Department of Mathematics and Statistics, University of Helsinki, Helsinki, Finland

**Keywords:** biomarkers, coronary heart disease, genomics, GWAS, polygenic risk scores

## Abstract

There are several established biomarkers for coronary heart disease (CHD), including blood pressure, cholesterol, and lipoproteins. It is of high interest to determine how a combined polygenic risk score (PRS) of CHD-associated biomarkers (BioPRS) can further improve genetic prediction of CHD. We developed CHDBioPRS, combining BioPRS with PRS of CHD in the UK Biobank and tested it on FinnGen. We found that BioPRS was clearly predictive of CHD and that CHDBioPRS improved the standard CHD PRS. The largest effect was observed with early onset cases in FinnGen, with HRs above 2 per standard deviation of CHDBioPRS.

## Highlights

• The novel BioPRS, constructed from combining statistically relevant CHD biomarkers, was clearly predictive of CHD in both the UK Biobank and FinnGen.
• CHDBioPRS, combining BioPRS with a standard CHD PRS, improved the prediction of the CHD PRS, with the largest effect size observed among the early onset cases.
• We observed similar HRs of CHDBioPRS for men and women.

## Visual Abstract

**Figure:**
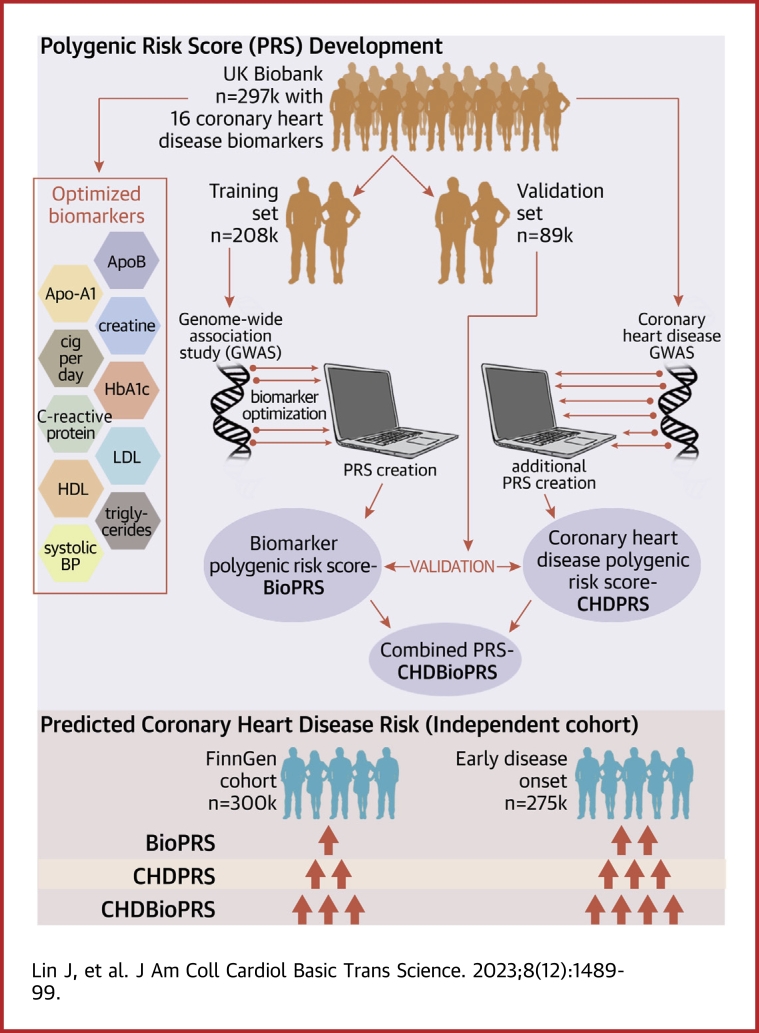

Coronary heart disease (CHD), a complex disease caused by a gradual build-up of fatty deposits in the arteries, is a major cause of death worldwide. In addition to family history, age, sex, smoking history, and levels of blood pressure, inflammation and lipoproteins are established biomarkers for CHD.^1^, ^2^, ^3^, ^4^ These risk factors are also used in clinical risk calculators to evaluate preventive therapies and strategies. Although clinical risk scores enable identification of some individuals at high risk,^5^, ^6^, ^7^ a large proportion of CHD cases are not detected by these scores, and the utility of clinical scores is limited for young adults^3^^,^^8^, ^9^, ^10^ and women.^11^^,^^12^

Genome-wide association studies (GWAS) involving large human genetic data sets have identified more than 100 loci statistically associated with CHD, mostly within populations of European descent.^13^, ^14^, ^15^, ^16^, ^17^, ^18^ These genetic discoveries together with the introduction of sophisticated statistical tools that incorporate linkage disequilibrium information, have greatly advanced risk prediction.^19^, ^20^, ^21^ Particularly, several studies have shown that inclusion of polygenic risk scores (PRS) improve CHD prediction and identification of high-risk groups.^13^^,^^22^, ^23^, ^24^ Because PRS are based on germline DNA, risk profiling can be conducted in early life when the individuals with the highest values of PRS are likely to benefit from an early adoption of preventive strategies.

The landmark study of PRS for common diseases conducted by Khera et al^23^ showed that a sizable portion of the population carry a polygenic CHD risk equivalent to known monogenic mutations conferring severalfold increased risk. This PRS, comprising more than 6 million single nucleotide polymorphisms (SNPs), was generated with the use of LDPred^19^ and has proven to be effective in validation sets across multiple populations while also performing favorably compared with other PRS^25^ composed of smaller numbers of variants.

Because PRS have proven to be successful for CHD prediction, it remains of high interest to systematically determine how a combined polygenic biomarker score (BioPRS) constructed with biomarkers associated with CHD can improve on the established CHDPRS. Recently, multi-PRS models, using 35 PRS from blood and urine biomarkers, have been shown to improve genetic risk prediction of common diseases such as type 2 diabetes and gout.^2^ While other existing studies have focused on combining several GWAS of CHD,^13^^,^^26^^,^^27^ our focus is on the combination of effects of known CHD-associated biomarkers into a single PRS and its integration with CHDPRS. Furthermore, because the current risk calculators do not work equally well for women as for men, it is of high importance to quantify the contribution of BioPRS within each sex.^28^ Another important goal is to predict a subgroup of CHD cases with an early onset of the disease.^29^

## Methods

**Figure 1:**
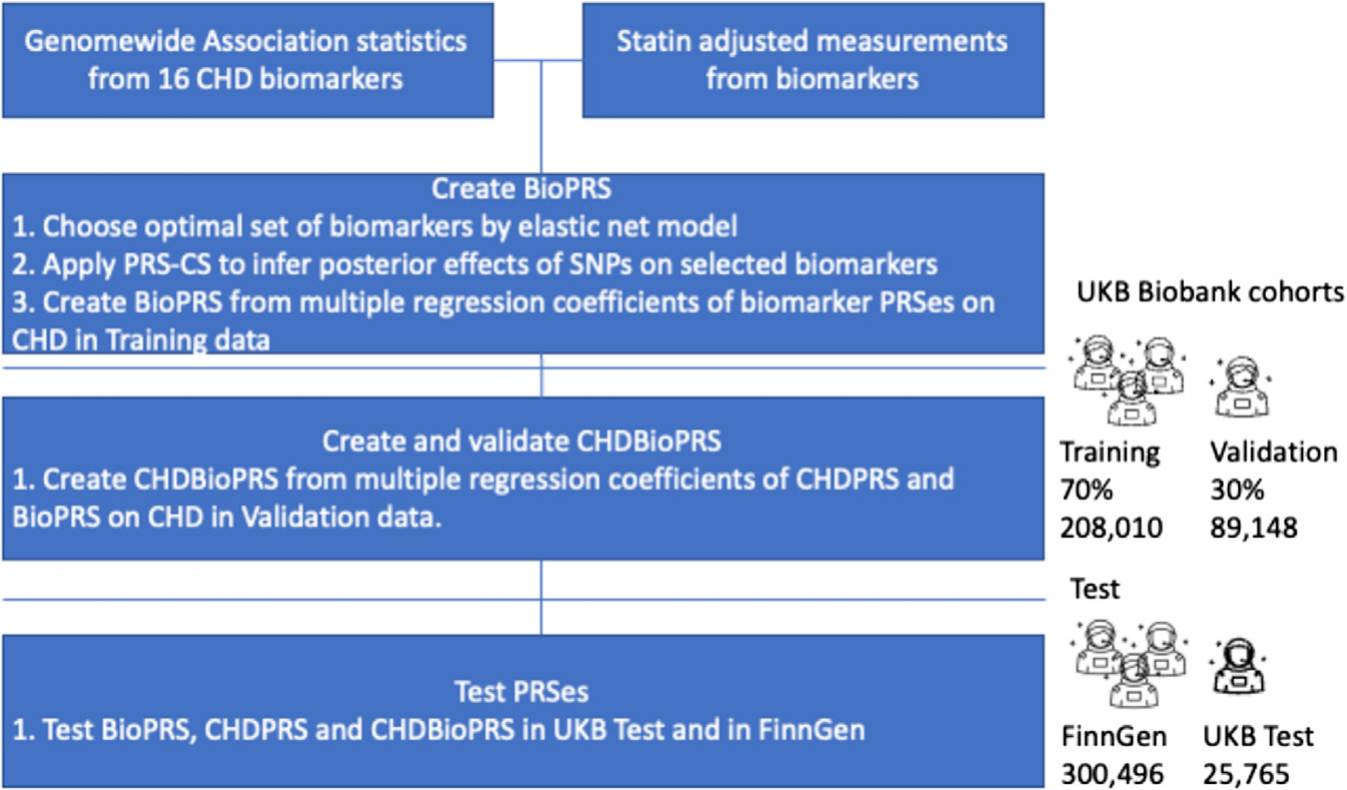
Study Design and Workflow CHD biomarkers: We identified CHD associated risk factors or biomarkers from UKB (Table 2, Supplemental Table 1). Genome-wide association study (GWAS) was performed on UKB genotype data (UKB (self-reported British White) Training and Validation combined), separately in females and males, for 16 biomarkers. Construction and testing of CHDBioPRS: We regressed CHD on the biomarkers in the UKB training data using elastic net Cox regression and retained 10 biomarkers. BioPRS is constructed by weighting each PRS by its coefficient in joint Cox regression model for CHD in UKB training data. CHDBioPRS is the sum of the standard CHDPRS and BioPRS where the weights are estimated from a Cox regression model predicting CHD within UKB validation data. Independent testing was performed on the FinnGen and the UKB Test (self-reported Non-British White) cohorts. CHD = coronary heart disease; UKB = UK Biobank.

The workflow of the study is presented in Figure 1.

### UK Biobank data

The design of UK Biobank (UKB) and the background of its participants have been reported previously.^22^^,^^30^ We restricted our analyses to samples that were of self-reported European ancestry to avoid potential spurious associations driven by allele frequency differences when including individuals from different ancestry backgrounds in our GWAS. We made use of 4 sets of UKB samples. First, our GWAS set contained 343,695 samples who were unrelated (pairwise kinship coefficients reported by UKB <0.044) and who self-reported “White British” as their ethnicity. Out of the GWAS set, 297,158 individuals had data on all 16 biomarkers (listed subsequently) and we split these into our UKB training (70%, 208,010 individuals) and UKB validation (30%, 89,148 individuals) sets. The split was done randomly by maintaining a constant CHD case ratio and sex ratio within each set. Finally, we also collected a UKB test set of 25,765 unrelated individuals with self-reported ethnicity as “White non-British” and biomarker measurements available. The UKB test set did not overlap with the UKB GWAS set, UKB training set, or UKB validation set.

### UKB CHD definition

The UKB CHD endpoint was defined as fatal or nonfatal myocardial infarction (MI), or coronary revascularization (percutaneous transluminal coronary angioplasty [PTCA] or coronary artery bypass grafting [CABG]). In detail, and consistently with previous studies,^23^^,^^31^ CHD cases were defined as having a heart attack diagnosed by a doctor or self-report of noncancer illness or operation including PTCA and CABG. In addition, coronary revascularization was assigned based on OPCS-4 coded procedure for CABG (K40.1-4, K41.1-4, and K45.1-5) or PTCA (K49.1-2, K49.8-9, K50.2, K75.1-4, and K75.8-9). MI cases from summary hospital episode statistics (ICD-10 and ICD-9 codes) were defined with ICD-10 I21, I22, I23, I24.1, and I25.2 and ICD-9 410, 411, and 412. We further merged the outcomes from the UKB MI algorithmically defined outcomes and dates. The UKB field codes of the CHD endpoint are listed in Table 1. We defined prevalent CHD cases as those who had CHD already when their blood sample was taken; other CHD cases were considered incident CHD cases. The age at onset in prevalent cases was determined by hospital episode data and self-reported age of onset, or for incident CHD cases by hospital or death records demonstrating disease onset after UKB enrollment.

**Table 1:** Sample Characteristics in UKB and FinnGen

	UKB	UKB Female	UKB Male	FinnGen	FinnGen Female	FinnGen Male
Sample size	297,158	158,382	138,776	300,496	169,508	130,988
Enrollment age, y	56.9 ± 7.99	56.7 ± 7.90	57.12 ± 8.09	53.88 ± 16.63	52.35 ± 16.96	55.86 ± 15.74
Incident CHD cases	5,011 (1.69)	1,353 (0.90)	3,658 (2.63)	13,623 (4.53)	3,652 (2.15)	9,971 (7.61)
Age at onset for incident cases, y^a^	65.13 ± 6.90	66.24 ± 6.95	64.92 ± 7.14	70.29 ± 10.80	72.56 ± 12.16	69.46 ± 10.13
Follow-up, y	10.01 ± 0.90	10.00 ± 0.89	10.04 ± 0.90	10.43 ± 8.96	9.22 ± 8.72	10.89 ± 9.01
All CHD cases^b^	16,803 (5.65)	3,762 (2.38)	12,896 (9.30)	30729 (10.22)	8,486 (5.00)	22,243 (16.98)
Age at onset, y	59.93 ± 9.43	61.41 ± 9.83	59.50 ± 9.26	65.37 ± 11.82	67.97 ± 12.50	64.37 ± 11.39

Values are mean ± SD or n (%).

a For incident coronary heart disease (CHD) cases, age at onset is after the blood sample was taken.

b CHD case endpoints were defined based on the following UKB (UK Biobank) fields (codes): myocardial infarction (MI) hospitalization: 41202 (ICD-10)/41203 (ICD-9); heart attack doctor diagnosed: 6150; percutaneous transluminal coronary angioplasty operation: 20004 (K49.1-2, K49.8-9, K50.2, K75.1-4, and K75.8-9); coronary revascularization: 41272 (K40.1-4, K41.1-4, and K45.1-5); and MI algorithm: 42000/42001 (date of onset).

### UKB biomarkers and GWAS

We identified 16 variables from UKB that have been previously reported to be associated with CHD (the UKB codes are listed in Table 2): high-density-lipoprotein (HDL) cholesterol, low-density-lipoprotein (LDL) direct, triglycerides (TRIG), apolipoprotein (Apo) A1, ApoB, diastolic blood pressure, systolic blood pressure (SBP), glycated hemoglobin (HbA_1c_), glucose, C-reactive protein (CRP), creatinine, lipoprotein(a), doctor-diagnosed diabetes, body mass index (BMI) and cigarettes per day (CPD). In addition, total cholesterol was calculated from the Friedewald formula^32^ as HDL + LDL + TRIG/2.2 in units of mmol/L. For simplicity, we use the term “biomarker” to refer to all of these variables even though some of them (namely, blood pressure, diabetes, BMI, and CPD) are not typical biomarkers measured from a blood or tissue sample.

**Table 2:** UKB Biomarkers Mean Levels After Adjustment for Statins

	UKB Code	Control Cases (Female)	Incident CHD Cases (Female)	Statin Adjustment^a^
Apolipoprotein A1,^b^ g/L	30630	1.52 ± 1.61	1.37 ± 1.51	/1.065364
Apolipoprotein B,^b^ g/L	30640	1.08 ± 1.07	1.14 ± 1.15	/0.721928
Body mass index, kg/m^2^	21001	27.31 ± 26.99	28.82 ± 28.52	
Cigarettes per day^b^	3456	1.05 ± 0.92	1.89 ± 2.19	
Creatinine^b^, μmol/L	30700	71.60 ± 63.98	75.38 ± 62.91	/1.0580718
C-Reactive protein,^b^ mg/L	30710	2.48 ± 2.61	2.89 ± 3.54	/1.2300281
Diabetes	2443	4.1% ± 3.1%	13.1% ± 10.3%	
Diastolic BP, mm Hg	4079	84.12 ± 82.24	87.09 ± 84.97	+10 (BP meds)
Glucose, mmol/L	30740	5.07 ± 5.03	5.37 ± 5.33	/1.028824
HbA_1c_,^b^ mmol/mol	30750	35.54 ± 35.41	38.0 ± 37.53	/1.0418022
HDL,^b^ mmol/L	30760	1.46 ± 1.60	1.22 ± 1.43	/1.053
Lipoprotein A, nmol/L		34.25 ± 35.17	38.59 ± 37.81	/1.101954
LDL,^b^ mmol/L	30780	3.78 ± 3.80	3.90 ± 4.04	/0.684
Systolic BP,^b^ mm Hg		140.8 ± 137.85	149.65 ± 148.04	+15 (BP meds)
Total cholesterol, mmol/L	NA^c^	6.06 ± 6.16	6.26 ± 6.56	
Triglycerides,^b^ mmol/L	30870	1.78 ± 1.58	2.18 ± 1.96	/0.874

BP = blood pressure; HDL = high-density lipoprotein; LDL = low-density lipoprotein; other abbreviations as in Table 1.

a Statin adjustment for statin users (16.2%) is done either by division (/) or addition (+) by the value given in the last column. Statin and blood pressure medicinal use was identified with the use of fields 20003, 6153, and 6177.

b Selected in optimal regularized model.

c Total cholesterol calculated from Friedewald formula of HDL + LDL + TRIG/2.2 in units of mmol/L.

Statin usage was identified from treatment medication (13 drugs: 1141146234, atorvastatin; 1141192414, crestor 10 mg tablet; 1140910632, eptastatin; 1140888594, fluvastatin; 1140864592, lescol 20 mg capsule; 1141146138, lipitor 10 mg tablet; 1140861970, lipostat 10 mg tablet; 1140888648, pravastatin; 1141192410, rosuvastatin; 1141188146, simvador 10 mg tablet; 1140861958, simvastatin; 1140881748, zocor 10 mg tablet; 1141200040, zocor heart-pro 10 mg tablet). Adjustment for statin usage^2^ was done for the following biomarkers by dividing the biomarker value with the given coefficient: HDL 1.053, LDL 0.684, TRIG 0.874, ApoA1 1.07, ApoB 0.722, lipoprotein(a) 1.102, glucose 1.029, CRP 1.230, creatinine 1.058, and HbA_1c_ 1.042. We also adjusted the subjects taking blood pressure reduction drugs by adding +15 to SBP and +10 to diastolic blood pressure. UKB statin and blood pressure drug codes are listed in Table 2, along with the statin and blood pressure–adjusted values of these biomarkers for the UKB training set.

### Genome-wide association study

Genome-wide association study (GWAS), using BOLT-LMM v2.3.2,^33^ was performed on UKB genotype data (release 3), separately in 183,130 women and 157,821 men, for the 16 biomarkers. We first regressed out sex, age, age-squared, and the top 10 principal components of genetic structure from the biomarkers and then applied rank-based inverse-normal transformation to the residuals.

### FinnGen

The design of the Finnish FinnGen^34^ project and participant backgrounds are presented in Table 1. The FinnGen test cohort contained 321,302 FinnGen data freeze 7 participants. The CHD case definition in FinnGen (I9_CHD) is consistent with our UKB CHD definition except that the FinnGen definition also includes samples with angina only (I20.0) as cases. Consequently, we removed the 2,989 angina-only cases from FinnGen, in addition to removal of 6,109 participants younger than 16 years old at enrollment.

### Biomarker model

Using the 16 CHD associated biomarkers for the UKB training data as predictors and incident CHD as outcome (and excluding prevalent CHD cases), we used penalized Cox proportional hazard models (glmnet R package) using the elastic net penalty (α = 0.50) with 20-fold cross-validation.^35^^,^^36^ The optimal model identified 10 biomarkers, which were selected for BioPRS construction (Table 2). As shown in Supplemental Figure 1, α = 0.25 (closer to ridge regression) produced the same optimal set as α = 0.50, and α = 0.75 (closer to lasso regression) further excluded ApoA1.

### Biomarker PRS

PRS with continuous shrinkage (CS)^21^ was run on each of the GWAS summary results of the selected 10 biomarkers. To account for linkage disequilibrium (LD), we used the 1000 Genomes^13^^,^^37^ project’s phase 3 European reference panel ,which resulted in LD-adjusted posterior effect sizes for 1,139,910 SNPs. The PRS for each biomarker was then computed as a sum over SNPs of the products of the individual’s genotype and the posterior effect size for the SNP with the use of PLINK2.0.^38^

### Weights of biomarker PRS on CHD

The PRS of the 10 selected biomarkers were used as predictors for incident CHD in a Cox proportional hazards model in the UKB training data without the prevalent CHD cases. According to this model, the hazard rate at age *t* depends on the predictors as follows:

(Equation 1) $$h\left(t\right)=h_{0}\left(t\right)\cdot \exp\left(b_{hdlPRS}x_{hdlPRS}+b_{ldlPRS}x_{ldlPRS}+b_{tgPRS}x_{tgPRS}+b_{apoaPRS}x_{apoaPRS}+b_{apobPRS}x_{apobPRS}+b_{cpdPRS}x_{cpdPRS}+b_{creaPRS}x_{creaPRS}+b_{cpdPRS}x_{cpdPRS}+b_{hba1cPRS}x_{hba1cPRS}+b_{sbpPRS}x_{sbpPRS}+b_{z}^{T}x_{z}\right)$$

Where *h*_*0*_*(t)* is the baseline hazard rate, *z* denotes the vector of covariate values (sex and the first 10 principal components of population structure), and each biomarker has coefficient *b*_biomarkerPRS_ that corresponds to a change in the logarithm of the hazard rate per one standard deviation of the biomarker PRS value.

We used the “coef” function from the “survival” library^39^ of R software to estimate the *b*_biomarkerPRS_ coefficients as previously recommended.^40^^,^^41^

### BioPRS

We combined the biomarker PRS into a score named BioPRS by standardizing (mean of 0 and SD of 1) the sum of the 10 biomarker PRS after multiplying each PRS by the beta-coefficient (b_i_) of the corresponding biomarker from formula (equation 1):

(Equation 2) $$BioPRS=standardize\left(\sum_{i=1}^{10}b_{i}\cdot {PRS}_{i}\right)$$

### CHDPRS

We generated a PRS for CHD (named CHDPRS) by applying PRS-CS^21^ to CHD GWAS reported by Nikpay et al^13^ using the European panel from the 1000 Genomes Project^37^ for LD reference. Our CHDPRS contained 1,087,715 SNPs. In addition, we compared our CHDPRS with “Khera PRS,” which is the PRS for CHD generated with the use of LDpred by Khera et al^23^ based on the same GWAS^13^ that we used to generate our CHDPRS.

### CHDBioPRS

CHDBioPRS was constructed from integration of BioPRS and CHDPRS. Weights of the 2 PRS (CHDPRS and mBioPRS) were estimated in the UKB validation set with the use of a Cox regression model with CHD as outcome:

(Equation 3) $$h\left(t\right)=h_{0}\left(t\right)\;\cdot \;\exp\left(c_{CHDPRS}x_{CHDPRS}+c_{BioPRS}x_{BioPRS}\right)$$

CHDBioPRS is the standardized sum of the CHDPRS and BioPRS multiplied by their weights from formula (equation 3):

(Equation 4) $$CHDBioPRS=standardize\left({c_{CHDPRS}\;CHDPRS}_{i}+c_{BioPRS}{BioPRS}_{i}\right)$$

In addition to the derivation above, a similar procedure was done also for men and women separately (biomarker selection using glmnet, biomarker weights in BioPRS using Cox regression, and combination of CHDPRS and BioPRS using another Cox regression).

### SCORE2

SCORE2^42^ is a prediction model for 10-year cardiovascular disease risk that uses information on age, total cholesterol, HDL, SBP, diabetes, and smoking. We calculated SCORE2 in our UKB data sets to give a comparison point for our PRS. We note that performance of SCORE2 may be overly optimistic in UKB, because the UKB data were used in the derivation of SCORE2. Because SCORE2 requires laboratory measurements, it cannot be applied in FinnGen, where those lab measurements are not available. We constructed combined predictors SCORE2 + CHDPRS and SCORE2 + CHDBioPRS by an approach similar to that described by equations (3) and (4).

### Early onset

We identified all individuals with early CHD onset (<55 years of age), and to further account for sex differences, we defined early CHD onset for women as <60 years of age and early CHD onset for men as <50 years of age.^43^

### Statistical analysis

All analyses were done with the use of R software.^36^^,^^39^^,^^44^, ^45^, ^46^ All scores were standardized to have a mean of 0 and a variance of 1. Cox proportional hazard models were used to estimate time until CHD ,with results presented as the HR with 95% CI and compared with the use of the likelihood ratio test statistic (LRT). In Cox regression, age was used as the time scale. When evaluating PRS, the CHD outcome variable included both incident and prevalent cases. In UKB, we excluded prevalent cases from the analysis when we selected relevant biomarkers and when we estimated the biomarker weights for generating BioPRS. In survival analyses of early onset cases, the cases with late onset were excluded and the control cases were censored at the upper limit of the early onset age.

C-Index,^47^ a metric for prediction concordance, and AUC with 95% CI were used to assess model discrimination. We also computed how much each biomarker PRS explains of the variance of the corresponding biomarker by using the adjusted *R*^2^ measure for the linear model where the biomarker PRS was the only predictor in the model. Net reclassification improvement (NRI) between different prediction models was obtained to determine how well the new model reclassifies patients compared with the previous model.

## Results

For our UKB GWAS set, we generated statin-adjusted GWAS results for 16 CHD-associated biomarkers. After accounting for withdrawals and missing biomarker measurements, we identified a total of 297,158 participants (53.3% female) comprising 16,658 CHD cases (22.6% female). We further split these data into training (70%) and validation (30%) sets while maintaining similar case and sex proportions. Our UKB test data set of 25,765 (56.4% female) unrelated individuals of “non-British White” self-identified ethnicity included 949 CHD cases (23.2% female). Our FinnGen test data contained 300,496 Finnish individuals (56.4% women; 10.4% CHD cases of which 27.6% were women). A combined PRS of CHD biomarkers (BioPRS) was derived across PRS of 10 biomarkers (Supplemental Table 1) (HDL, LDL, TRIG, total cholesterol, systolic blood pressure, CPD, HbA_1c_, CRP, creatinine, ApoB, ApoA1) by combining the individual biomarker PRS generated with PRS-CS on 1,106,191 SNPs. We validated in UKB test data that this BioPRS (C-index: 0.781; SE: 0.007) performed at least as well as a corresponding PRS constructed from all 16 biomarkers (C-index: 0.778; SE: 0.007). BioPRS was further integrated with the CHDPRS constructed from 6,630,150 variants from a GWAS^13^ involving 184,305 participants of European ancestry to yield CHDBioPRS. We also compared our CHDPRS to the Khera PRS^23^ and found that our CHDPRS gave a similar result in UKB training data (HR: 1.64 [95% CI: 1.61-1.67]; compared with HR: 1.62 (95% CI: 1.59-1.64) (Supplemental Table 2). CHDBioPRS is approximately normally distributed and, on average, higher in CHD cases relative to the control cases (mean difference: 0.518 SD; 95% CI: 0.490-0.546) (Supplemental Figure 2).

Table 3 presents the results from UKB data sets and FinnGen. In UKB, results from the training set are similar to results from the validation and test sets, which suggests that the model is not overfitting in the training data. In all 3 UKB data sets, we observed the same pattern, where BioPRS itself is clearly predictive of CHD (HR estimates per SD vary from 1.42 to 1.45), CHDPRS on its own is more predictive than BioPRS (HR estimates vary from 1.62 to 1.78), and CHDBioPRS is the most predictive (HRs vary from 1.73 to 1.88). For C-index, *z*-scores, and AUC metrics, see Supplemental Tables 2 to 4. When the scores are applied in FinnGen (Supplemental Table 5), the pattern was repeated, where BioPRS is clearly predictive and improves the prediction by CHDPRS when combined with CHDPRS into CHDBioPRS. Overall, the HRs in FinnGen are smaller than in UKB for all PRS.

**Table 3:** HRs (With 95% CIs) From Cox Regression Model of 3 Different PRS on Incident CHD

	BioPRS	CHDPRS	CHDBioPRS
UKB training	1.45 (1.42-1.48)	1.64 (1.61-1.67)	1.76 (1.72-1.79)
UKB validation	1.42 (1.38-1.46)	1.62 (1.57-1.67)	1.73 (1.68-1.78)
UKB test	1.45 (1.36-1.55)	1.78 (1.67-1.91)	1.88 (1.76-2.01)
UKB test women	1.53 (1.34-1.75)	1.72 (1.50-1.98)	1.86 (1.62-2.14)
UKB test men	1.42 (1.32-1.53)	1.72 (1.60-1.85)	1.84 (1.71-1.98)
UKB test early onset	1.60 (1.43-1.78)	1.90 (1.69-2.13)	2.07 (1.85-2.32)
FinnGen	1.27 (1.26-1.29)	1.57 (1.55-1.60)	1.60 (1.58-1.62)
FinnGen women	1.27 (1.24-1.30)	1.53 (1.50-1.57)	1.56 (1.53-1.60)
FinnGen men	1.27 (1.25-1.29)	1.58 (1.55-1.60)	1.61 (1.59-1.63)
FinnGen early onset	1.51 (1.47-1.55)	2.01 (1.95-2.07)	2.10 (2.04-2.16)

BioPRS is combination of PRS of 10 CHD-related biomarkers, CHDPRS is a standard PRS for CHD, and CHDBioPRS combines BioPRS and CHDPRS. Early onset was defined as CHD before 55 years of age. C-index, AUC, and *P* values are in Supplemental Tables 2 to 7, likelihood ratio statistics are in Supplemental Table 18, and SCORE2-related statistics are in Supplemental Table 19.

PRS = polygenic risk score; other abbreviations as in Table 1.

When comparing the CHD risk between the top 5% of the PRS distribution and the rest, we observed larger HRs for CHDBioPRS (UKB test: 4.16 [95% CI: 3.09-5.60], FinnGen: 4.04 [95% CI: 3.76-4.35]) than for CHDPRS (UKB test: 3.56 [95% CI: 2.62-4.86], FinnGen: 3.84 [95% CI: 3.56-4.14]. HRs for other percentiles along with their AUCs are listed in Supplemental Table 6 for UKB Test and Supplemental Table 7 for FinnGen.

**Figure 2:**
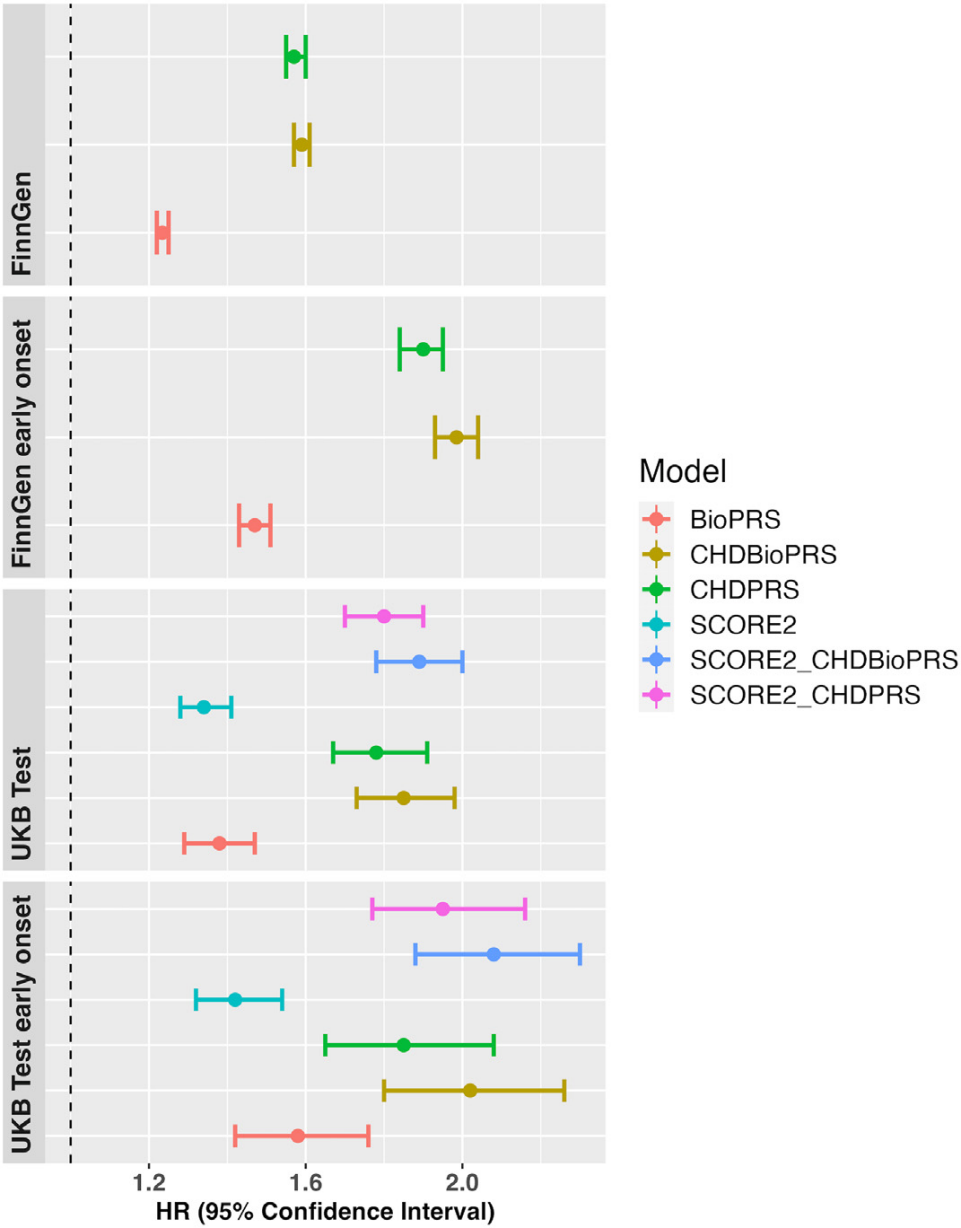
CHD Prediction Using PRSes in FinnGen and UKB Test Samples: Both Cohorts Are Disjoint From the Samples Used in Construction of BioPRS and CHDBioPRS (Early Onset = Onset <55 Years of Age) CHD = coronary heart disease; PRS = polygenic risk score; UKB = UK Biobank.

We next studied the PRS in cases with early CHD onset (<55 years of age). For UKB test data, we had 325 cases (20.6% female) and for FinnGen 5,965 cases (21.4% female). For both cohorts, we observed higher HRs for early onset cases than for all cases and again we saw HRs growing when using CHDBioPRS (UKB test: 2.07 [95% CI: 1.85-2.32], FinnGen: 2.10 [95% CI: 2.04-2.16]) instead of CHDPRS (UKB test: 1.90 [95% CI: 1.69-2.13], FinnGen: 2.01 [95% CI: 1.95-2.07] (Figure 2, Table 3; see also Supplemental Table 6 for UKB test and Supplemental Table 7 for FinnGen). Among all CHD cases, the CHDBioPRS values peaked for cases with an onset at around 40 years of age (Supplemental Figure 3).

It is known that CHD incidence and biomarker associations vary between sexes.^3^^,^^10^ Therefore, we stratified UKB data and FinnGen data by sex. In both sexes, the regularized optimal models built in UKB training data consisted of the same 8 biomarkers (HDL, LDL, TRIG, SBP, CPD, HbA_1c_, CRP, and ApoB). Using these biomarkers, we refitted the models in each sex separately and derived corresponding scores (BioPRS and CHDBioPRS). The CHD HR had similar dynamics between CHDPRS and CHDBioPRS compared with the full cohort (Table 3, Supplemental Tables 8 to 11). Among FinnGen women with onset before 60 years of age, the HRs were 1.82 (95% CI: 1.74-1.90) for CHDPRS and 1.90 (95% CI: 1.81-1.99) for CHDBioPRS (Supplemental Table 12), and for FinnGen men with onset before 50 years of age, the HRs were 2.17 (95% CI: 2.07-2.27) for CHDPRS and 2.26 (95% CI: 2.16-2.36) for CHDBioPRS (Supplemental Table 13).

We observed that CHDBioPRS provided improved prediction in terms of HR, C-index, and LRT compared with SCORE2, a prediction algorithm based only on measured risk factors, but that a combination of SCORE2 and CHDBioPRS gave the best performance (Figure 2, Supplemental Tables 18 and 19). In evaluating NRI, we found that CHDBioPRS improved NRI over CHDPRS (Supplemental Table 16) by 0.239 for UKB test, 0.301 for UKB test early onset, 0.101 for FinnGen, and 0.139 for FinnGen early onset. Finally, we also found that CHDBioPRS improved LRT compared with CHDPRS in both UKB test and FinnGen cohorts (Supplemental Figure 4, Supplemental Table 18).

## Discussion

In an analysis of more than 600,000 participants involving 2 nationwide study cohorts, UKB and FinnGen, we showed that combining a biomarker PRS derived from 10 CHD-associated biomarkers with a standard PRS for CHD improved the prediction of CHD. This improvement in prediction was largest for early onset CHD for both men and women.

Our BioPRS compared well with the recently published multi-PRS score by Sinnott-Armstrong et al,^2^ which was derived from 35 UKB biomarkers. Their multi-PRS score was reported to have an HR of 1.19 (95% CI: 1.17-1.22) in FinnGen (data freeze 3) for MI, while our BioPRS achieved an HR of 1.29 (95% CI: 1.27-1.31) using the same endpoint and same covariates in the more recent and larger data freeze 7 of FinnGen. In addition, when Sinnott-Armstrong et al combined their multi-PRS with a standard PRS for CHD in FinnGen, they reported an HR of 1.50 (95% CI: 1.46-1.53), while our CHDBioPRS achieved an HR of 1.60 (95% CI: 1.58-1.62) in FinnGen (data freeze 7). Similarly, a MetaGRS score from Inouye et al,^26^ which combined 3 genetic risk scores (GRS46K,^22^ another score based on 202 significant genetic variants from CARDIOGRAMplusC4D,^17^ and a genome-wide score based on the same GWAS^13^), reported an HR of 1.71 (95% CI: 1.68-1.73) when tested on UKB, while our CHDBioPRS yielded an HR of 1.88 (95% CI: 1.75-2.01) in our UKB test data.

There were clear differences in effect sizes between UKB and FinnGen. These differences could in part relate to differences in sample ascertainment procedures and genetic background. The UKB participants are known to be healthier than the general population,^48^ and therefore the relative contribution of genetics to their disease risk may be larger, whereas the FinnGen participants are recruited through their contacts with the Finnish health care system. In addition, the FinnGen participants are on average 5 years older than the UKB participants, and the CHD case rate in FinnGen is nearly double that of UKB (10.2% vs 5.6%). Differences in performance of PRS are known to exist even between populations of European ancestry.^49^ In our study, the biomarker GWAS effect sizes and LD information used in creating PRS were derived in UKB or from other non-Finnish European populations, which could lead to better predictive power of PRS in UKB compared with Finnish data.^50^ For our CHDPRS, the effect sizes were taken from a large GWAS meta-analysis^13^ that may have included some of our FinnGen test samples. However, because our CHDPRS performed better in UKB than in FinnGen, we do not expect this potential overlap to have caused serious overfitting in our FinnGen test data.

Both sex-specific CHDBioPRS were constructed from the same set of 8 biomarkers (HDL, LDL, TRIG, SBP, CPD, HbA_1c_, CRP, and ApoB) and achieved prediction improvements compared with the standard CHD PRS. Importantly, we observed fairly similar HR estimates for men and women, which is in contrast with existing CHD clinical scores, such as QRISK/QRISK2 which is known to underestimate the CHD risks in women.^51^, ^52^, ^53^, ^54^ We also observed larger HRs for early onset cases than all cases, indicating that our PRS are also informative about age at onset.

Throughout the analyses, we saw that prediction using CHDBioPRS was statistically strongly favored over that from CHDPRS when measured by likelihood ratio. Similarly, we saw that CHDBioPRS led to higher performance compared with SCORE2,^42^ which is a predictor based on traditional risk factor measurements, and the combination of CHDBioPRS and SCORE2 performed best. Thus, when both genetic data and risk factor measurements are available, the combination of the two may be beneficial. An important caveat here is that because we were able to compute SCORE2 only on UKB, and UKB data have been used in derivation of the SCORE2 algorithm, our SCORE2 predictions in UKB data may be too optimistic.

### Study Limitations

Disease risk prediction using multiple biomarkers and genome-wide set of genetic variants is a very high-dimensional problem and therefore adding more sparsity to the model building could improve the risk prediction. For example, one could attempt to use, for each genomic region separately, only a relevant subset of biomarkers.^55^ Furthermore, genomic regions could be prioritized, for example, by curated CHD molecular pathways^56^ including known lipid-associated genomic regions.^57^

The present study is limited to individuals with European ancestry. Given recent discoveries about imperfect transferability of PRS between populations,^58^^,^^59^ training of an optimal CHDBioPRS for non-European ancestries would require appropriate training data from those other ancestries. This is also important because the potential to use genetic scores to identify high-risk individuals from birth could exacerbate the health differences between individuals with European ancestry and others until there is a broader inclusion of underserved ethnicities in research, particularly in multiethnic countries such as the UK and the U.S.^60^

An approach similar to our BioPRS could also improve prediction of other complex diseases, such as type 2 diabetes and breast cancer, with established PRS and known heritable risk factors.^59^

## Conclusions

The integration of biomarker PRS improves on the standard PRS for prediction of CHD, where the gain was largest among early onset CHD cases. This study strengthens the evidence for genome-based CHD prediction and quantifies the interplay between standard CHD PRS and PRS of biomarkers associated with CHD.

### Ethics Statement and Methods

Patients and control subjects in FinnGen provided informed consent for biobank research, based on the Finnish Biobank Act. Alternatively, separate research cohorts, collected before the Finnish Biobank Act came into effect in September 2013 and the start of FinnGen in August 2017, were collected based on study-specific consents and later transferred to the Finnish biobanks after approval by the Finnish Medicines Agency (Fimea), the national supervisory authority for welfare and health. Recruitment protocols followed the biobank protocols approved by Fimea. The Coordinating Ethics Committee of the Hospital District of Helsinki and Uusimaa statement no. for the FinnGen study is HUS/990/2017.

The FinnGen study was approved by the Finnish Institute for Health and Welfare (permit nos. THL/2031/6.02.00/2017, THL/1101/5.05.00/2017, THL/341/6.02.00/2018, THL/2222/6.02.00/2018, THL/283/6.02.00/2019, THL/1721/5.05.00/2019, THL/1524/5.05.00/2020, and THL/2364/14.02/2020), the Digital and Population Data Service Agency (permit nos. VRK43431/2017-3, VRK/6909/2018-3, and VRK/4415/2019-3), the Social Insurance Institution (permit nos. KELA 58/522/2017, KELA 131/522/2018, KELA 70/522/2019, KELA 98/522/2019, KELA 138/522/2019, KELA 2/522/2020, and KELA 16/522/2020), Findata (THL/2364/14.02/2020) and Statistics Finland (permit nos. TK-53-1041-17 and TK/143/07.03.00/2020 [earlier TK-53-90-20]).

The Biobank Access Decisions for FinnGen samples and data used in FinnGen data freeze 7 include THL Biobank BB2017_55, BB2017_111, BB2018_19, BB_2018_34, BB_2018_67, BB2018_71, BB2019_7, BB2019_8, BB2019_26, and BB2020_1, Finnish Red Cross Blood Service Biobank 7.12.2017, Helsinki Biobank HUS/359/2017, Auria Biobank AB17-5154 and amendment no. 1 (August 17, 2020), Biobank Borealis of Northern Finland 2017_1013, Biobank of Eastern Finland 1186/2018 and amendment 22 § /2020, Finnish Clinical Biobank Tampere MH0004 and amendments 21.02.2020 and 06.10.2020, Central Finland Biobank 1-2017, and Terveystalo Biobank STB 2018001.

Perspectives **COMPETENCY IN MEDICAL KNOWLEDGE:** CHD is a leading cause of death, and there is an increased need for better genetic prediction, especially for high-risk individuals. A standard PRS for CHD has already been shown to improve prediction. A new PRS, constructed by combining the CHD PRS with PRS of statistically relevant CHD risk factors, can further improve genetic prediction, particularly for high-risk individuals. The identification of individuals with the highest risk of CHD can assist clinicians in their decision making.

**TRANSLATIONAL OUTLOOK:** Additional research is needed to establish the clinical benefit of the novel PRS, which includes information from multiple CHD-associated biomarkers, providing a more individualized prediction for CHD.

## Funding Support and Author Disclosures

This work was supported by the Academy of Finland (grant nos. 325999 to Dr Lin, 331671 to Dr Mars, 312076 and 336825 to Dr Pirinen, and 285380 and 312062 to Dr Ripatti) and the Sigrid Juselius Foundation (to Drs Pirinen and Ripatti). Dr Lin was also supported by GEMMA (H2020-SC1-BHC-03-2018, project ID 825033). All other authors have reported that they have no relationships relevant to the contents of this paper to disclose.
