# Supplementary material for: Integration of Biomarker Polygenic Risk Score Improves Prediction of Coronary Heart Disease
Source: JACC Basic Transl Sci. 2023 Oct 4;8(12):1489–99. doi: 10.1016/j.jacbts.2023.07.006 (PMC10774750; doi:10.1016/j.jacbts.2023.07.006)
Supplement: Supplemental Appendix [file mmc1.pdf]

Supplemental Appendix.

Supplemental figures

Supplemental Figure 1 – p.2
Supplemental Figure 2 – p.3
Supplemental Figure 3 – p.4
Supplemental Figure 4 – p.5

Supplemental tables

Supplemental Table 1 – p.6
Supplemental Table 2 – p.7
Supplemental Table 3 – p.8
Supplemental Table 4 – p.9
Supplemental Table 5 – p.10
Supplemental Table 6 – p.11
Supplemental Table 7 – p.12
Supplemental Table 8 – p.13
Supplemental Table 9 – p.14
Supplemental Table 10 – p.15
Supplemental Table 11 – p.16
Supplemental Table 12 – p.17
Supplemental Table 13 – p.18
Supplemental Table 14 – p.19
Supplemental Table 15 – p.20
Supplemental Table 16 – p.21
Supplemental Table 17 – p.22
Supplemental Table 18 – p.23
Supplemental Table 19 – p.24

A. Glmnet (elastic net regression) with an alpha value of 0.50 found 10 biomarkers in its optimal model (lambda.1SE setting), as indicated by the plot's right vertical dashed line and the selected inversed normalize (*_int) biomarker predictors are shown on the right. These same 10 biomarkers were found to be the optimal model using alpha value of 0.25. A lambda.min setting for both 0.25 and 0.50 lambda values selected all 16 biomarkers (as indicated by the left vertical dashed line) is not selected due to overfitting.

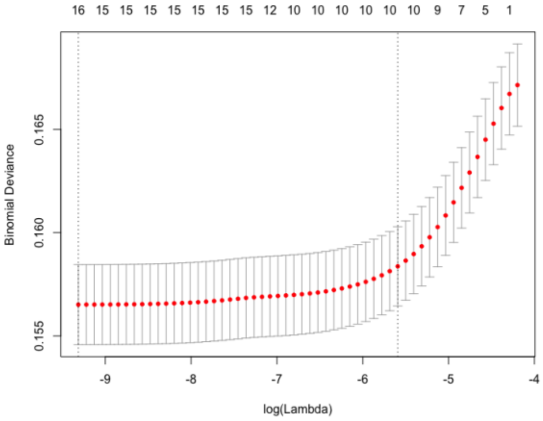

adj_hdl_int	-0.16225960
adj_ldl_int	0.11438159
adj_tg_int	0.04423273
adj_tc1_int	.
bmi_int	.
ApoB_int	0.08207446
ApoA1_int	-0.02131672
gluc_int	.
lpa_int	.
t2d_int	.
sbp_int	0.25952935
dbp_int	.
crea_int	0.20981747
CRP_int	0.03610340
hba1c_int	0.06288727
cigs_perday_int	0.08135247

B. Glmnet with an alpha value of 0.75 found 9 biomarkers (ApoA1 excluded). To balance sensitivity and fit, we select the 10 biomarker optimal models, selected by models with 0.50 and 0.25 alpha values.

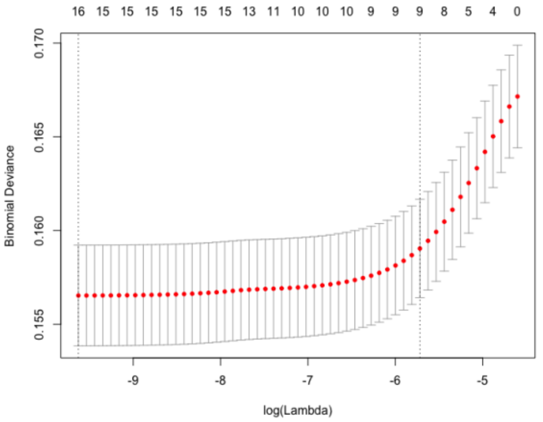

adj_hdl_int	-0.17227396
adj_ldl_int	0.09088489
adj_tg_int	0.03217825
adj_tc1_int	.
bmi_int	.
ApoB_int	0.08156334
ApoA1_int	.
gluc_int	.
lpa_int	.
t2d_int	.
sbp_int	0.24881371
dbp_int	.
crea_int	0.18067260
CRP_int	0.01205109
hba1c_int	0.04223650
cigs_perday_int	0.06876118

Supplemental Figure 1: Glmnet (elastic net regression) biomarker selection

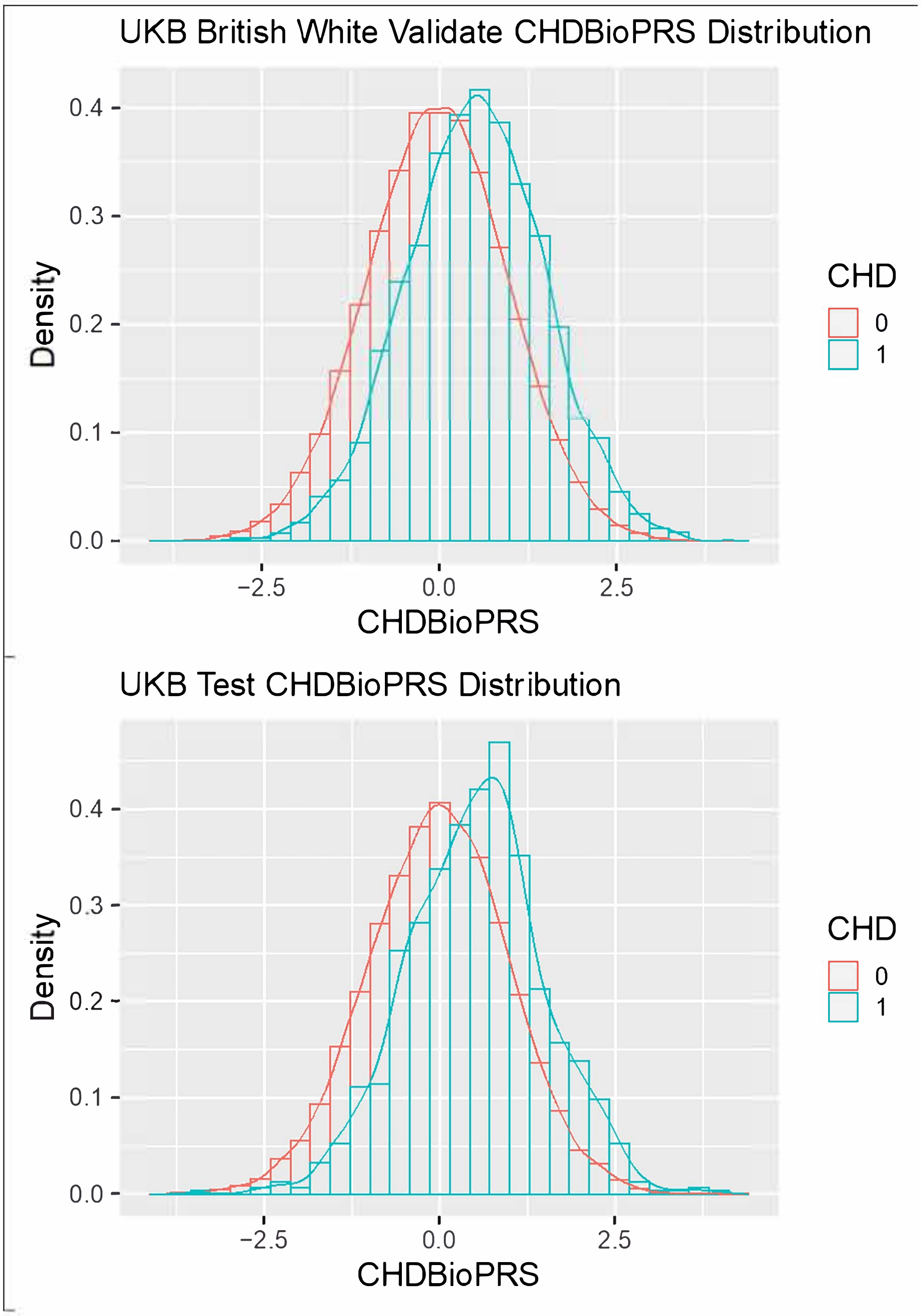

Figure S2 CHDBioPRS score distribution in cases and controls. CHDBioPRS scores for UKB Training (not shown), Validation (Top) and Test (Bottom) cohorts, are approximately normally distributed and on average, higher in CHD cases (blue) than in controls (red).

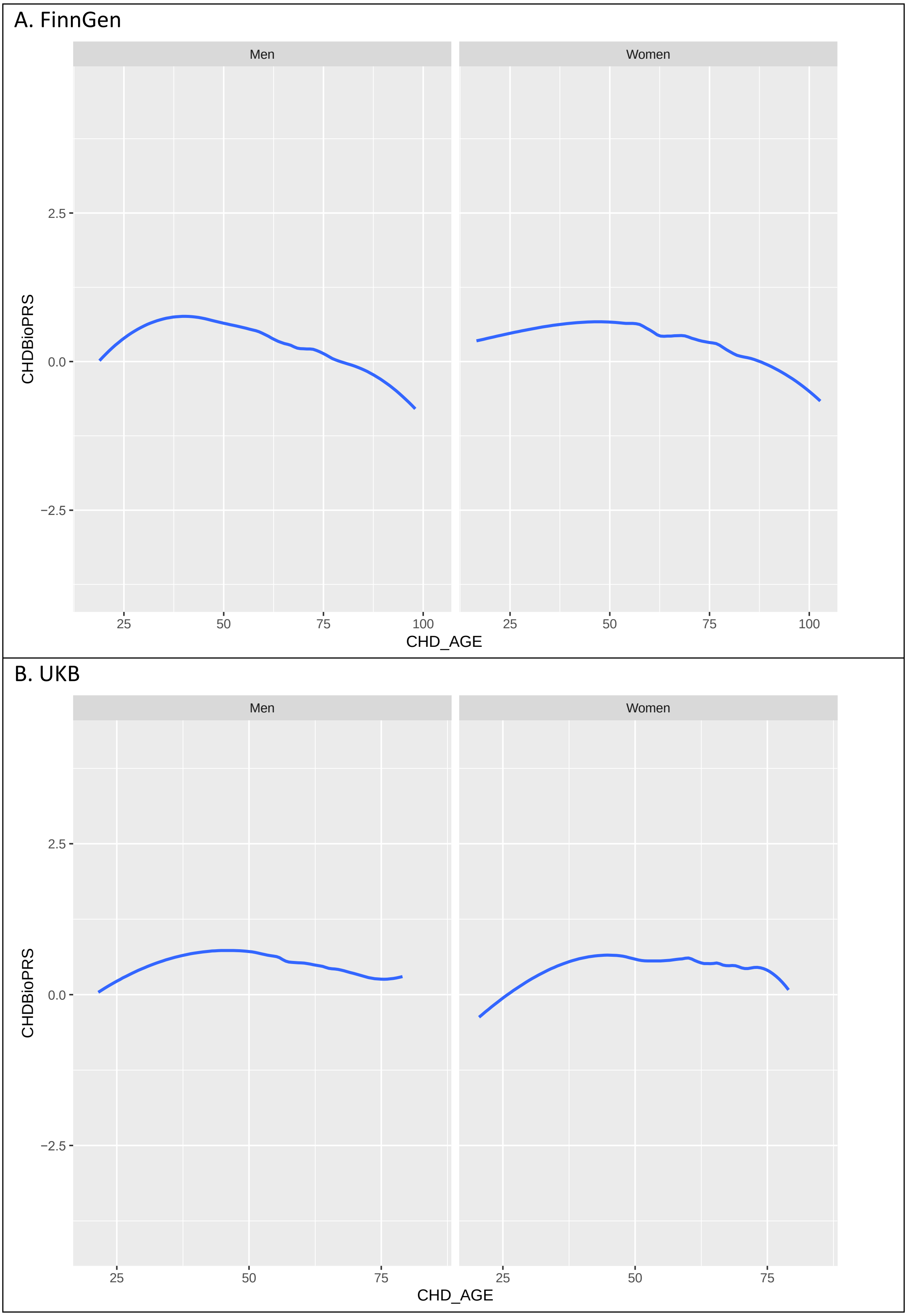

Supplemental Figure 3. LOESS regression curves of the mean CHDBioPRS scores and onset age separated by sex and cohorts (A) FinnGen and (B) UKB.

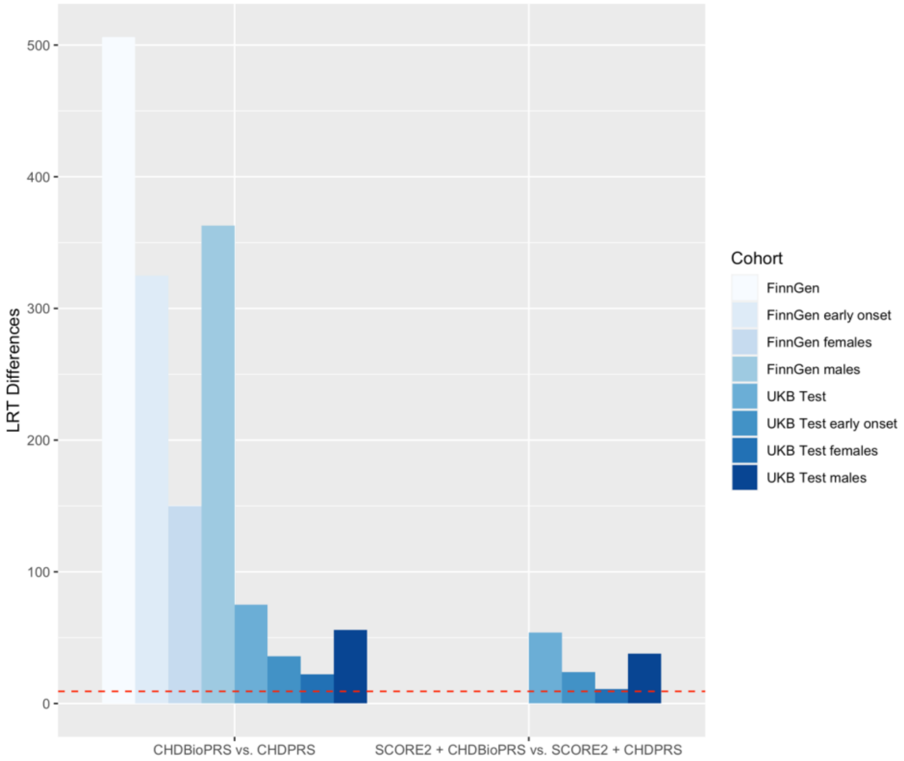

Figure S4. LRT differences between CHDBioPRS and CHDPRS on different data sets. On right, a comparison with SCORE2 integrated in both PRS in the cox regression model. On the left, a comparison without SCORE2 integrated into PRS. Across all cohorts and models, the integration of BioPRS into CHDPRS improves the likelihood and the improvement is always > 9.21 (indicated by the dashed red line) implying over 100-fold increase in the likelihood value. (Notes: Y-axis is on logarithmic scale; SCORE2 cannot be calculated for the FinnGen study due to a lack of measurements needed for SCORE2.)

Supplemental Table 1: Hazard ratio and variance explained of the PRSes of 10 biomarkers selected by the elastic net analysis

**Biomarker**	**HR (95% CI) for CHD per 1 SD of PRS**						**Variance explained (R-squared)**		
	**UKB Test**	**UKB Test Female**	**UKB Test Male**	**FinnGen**	**FinnGen Female**	**FinnGen Male**	**UKB Test**	**UKB Test Female**	**UKB Test Male**
**Apo-A1**	0.89 (0.83-0.95)	0.88 (0.77-1.00)	0.89 (0.83-0.96)	0.91 (0.90-0.92)	0.92 (0.90-0.94)	0.91 (0.90-0.92)	0.097	0.104	0.136
**ApoB***	1.32 (1.24-1.41)	1.33 (1.16-1.52)	1.32 (1.23-1.42)	1.18 (1.17-1.20)	1.14 (1.12-1.17)	1.20 (1.18-1.22)	0.165	0.170	0.161
**CPD***	1.12 (1.05-1.19)	1.14 (1.00-1.30)	1.11 (1.03-1.19)	1.05 (1.03-1.06)	1.03 (1.01-1.06)	1.05 (1.04-1.07)	0.009	0.008	0.01
**CREA**	1.00 (0.93-1.06)	0.93 (0.81-1.06)	1.02 (0.95-1.09)	0.96 (0.95-0.97)	0.97 (0.95-1.00)	0.96 (0.95-0.97)	0.081	0.129	0.099
**C-Reactive Protein***	1.02 (0.96-1.08)	1.07 (0.93-1.22)	1.00 (0.93-1.08)	1.01 (0.99-1.02)	1.02 (1.00-1.05)	1.00 (0.99-1.02)	0.106	0.120	0.089
**HbA1c***	1.09 (1.02-1.16)	1.01 (0.89-1.16)	1.12 (1.04-1.20)	1.06 (1.04-1.07)	1.06 (1.04-1.09)	1.05 (1.04-1.07)	0.129	0.129	0.130
**HDL***	0.87 (0.82-0.93)	0.84 (0.74-0.96)	0.88 (0.82-0.95)	0.90 (0.89-0.91)	0.91 (0.88-0.93)	0.90 (0.89-0.92)	0.129	0.171	0.156
**LDL***	1.30 (1.22-1.38)	1.30 (1.14-1.50)	1.29 (1.20-1.39)	1.17 (1.16-1.18)	1.13 (1.11-1.15)	1.19 (1.17-1.20)	0.156	0.162	0.149
**SBP***	1.26 (1.18-1.34)	1.34 (1.17-1.53)	1.23 (1.15-1.33)	1.19 (1.17-1.20)	1.22 (1.19-1.25)	1.17 (1.16-1.19)	0.073	0.077	0.071
**TRIG***	1.19 (1.12-1.27)	1.29 (1.13-1.47)	1.16 (1.08-1.25)	1.09 (1.08-1.10)	1.09 (1.07-1.10)	1.09 (1.08-1.11)	0.103	0.110	0.111

*Selected by elastic net sex specific models

Adjusted for age, sex and the first 10 principal components of genetic structure.

CHD=Coronary heart disease, PRS=Polygenic risk score, HR=Hazard ratio, CI=confidence interval, UKB=UK Biobank, CPD=Cigarettes per day, CREA=creatinine, HbA1c=Hemoglobin-A1c, HDL=High-density lipoprotein, LDL=Low-density lipoprotein, SBP=Systolic blood pressure, TRIG=Triglyceride

Supplemental Table 2: Results from UKB Training data for quantile levels of PRSes

Model	Quantile	HR for CHD (95% CI)	C-Index (SE)	AUC (95% CI)
**Baseline model**				.759 (.754-.763)
**BioPRS**		1.43 (1.41-1.46)	.763 (.002)	.775 (.771-.780)
	Q80+	1.85 (1.77-1.92)	.755 (.002)	.768 (.764-.772)
	Q90+	1.92 (1.82-2.01)	.752 (.002)	.765 (.761-.769)
	Q95+	1.96 (1.84-2.09)	.750 (.002)	.763 (.758-.766)
	Q99+	2.07 (1.81-2.38)	.747 (.002)	.760 (.755-.764)
**CHDPRS**		1.64 (1.61-1.67)	.776 (.002)	.787 (.783-.791)
	Q80+	2.30 (2.21-2.39)	.764 (.002)	.776 (.772-.780)
	Q90+	2.46 (2.35-2.57)	.759 (.002)	.772 (.768-.776)
	Q95+	2.70 (2.55-2.86)	.755 (.002)	.768 (.764-.772)
	Q99+	3.66 (3.29-4.06)	.750 (.002)	.762 (.758-.767)
**CHDBioPRS**		1.75 (1.72-1.78)	.783 (.002)	.795 (.791-.799)
	Q80+	2.56 (2.46-2.66)	.769 (.002)	.781 (.777-.785)
	Q90+	2.77 (2.65-2.89)	.763 (.002)	.776 (.771-.779)
	Q95+	2.98 (2.82-3.15)	.757 (.002)	.770 (.766-.774)
	Q99+	3.66 (3.30-4.07)	.750 (.002)	.763 (.759-.767)

Baseline model includes age, sex and the first 10 principal components of genetic structure. Other models add a PRS to the baseline model. For each PRS, the HR is for CHD and per 1 SD of PRS. For each quantile (Q), the HR is for the indicator of the risk group vs. all the others. For example, Q80+ compares the individuals with the highest 20% of PRSes to the remaining 80%. C-Index is from Cox regression model. AUC is computed from a roc (pROC package) call using the prediction results of a CHD logistic regression model adjusted for the baseline parameters.

UKB=UK Biobank, PRS=Polygenic risk score, CHD=Coronary heart disease, HR=Hazard ratio, CI=Confidence interval, SD=Standard deviation, Q=Quantile, SE=Standard error, AUC=Area under the curve, BioPRS=PRS constructed from selected biomarkers, CHDPRS=standard CHD PRS, CHDBioPRS=Risk score integrating BioPRS and CHDPRS

Supplemental Table 3: Results from UKB Validation data for quantile levels of PRSes

Model	Quantile	HR for CHD (95% CI)	C-Index (SE)	AUC (95% CI)
**Baseline model**				.762 (.756-.768)
**BioPRS**		1.41 (1.37-1.45)	.763 (.003)	.777 (.771-.783)
	Q80+	1.74 (1.64-1.85)	.755 (.003)	.770 (.764-.776)
	Q90+	1.82 (1.69-1.96)	.753 (.003)	.768 (.761-.774)
	Q95+	1.92 (1.74-2.12)	.750 (.003)	.765 (.759-.772)
	Q99+	2.17 (1.78-2.66)	.748 (.003)	.763 (.757-.770)
**CHDPRS**		1.62 (1.57-1.67)	.776 (.003)	.790 (.784-.796)
	Q80+	2.22 (2.09-2.35)	.764 (.003)	.778 (.772-.784)
	Q90+	2.43 (2.27-2.61)	.761 (.003)	.775 (.769-.781)
	Q95+	2.65 (2.43-2.89)	.757 (.003)	.772 (.765-.778)
	Q99+	3.50 (2.96-4.13)	.751 (.003)	.766 (.760-.772)
**CHDBioPRS**		1.72 (1.67-1.77)	.783 (.003)	.795 (.789-.801)
	Q80+	2.48 (2.34-2.63)	.769 (.003)	.783 (.777-.789)
	Q90+	2.63 (2.46-2.81)	.763 (.003)	.777 (.771-.784)
	Q95+	2.80 (2.57-3.06)	.758 (.003)	.772 (.767-.779)
	Q99+	3.98 (3.40-4.68)	.751 (.003)	.767 (.760-.773)

Baseline model includes age, sex and the first 10 principal components of genetic structure. Other models add a PRS to the baseline model. For each PRS, the HR is for CHD and per 1 SD of PRS. For each quantile (Q), the HR is for the indicator of the risk group vs. all the others. For example, Q80+ compares the individuals with the highest 20% of PRSes to the remaining 80%. C-Index is from Cox regression model. AUC is computed from a roc (pROC package) call using the prediction results of a CHD logistic regression model adjusted for the baseline parameters.

UKB=UK Biobank, PRS=Polygenic risk score, CHD=Coronary heart disease, HR=Hazard ratio, CI=Confidence interval, SD=Standard deviation, Q=Quantile, SE=Standard error, AUC=Area under the curve, BioPRS=PRS constructed from selected biomarkers, CHDPRS=standard CHD PRS, CHDBioPRS=Risk score integrating BioPRS and CHDPRS

Supplemental Table 4: Results from UKB Test data for quantile levels of PRSes

Model	Quantile	HR for CHD (95% CI)	C-Index (SE)	AUC (95% CI)
**Baseline model**				.777 (.763-.791)
**BioPRS**		1.45 (1.36-1.54)	.786 (.007)	.794 (.780-.807)
	Q80+	1.92 (1.67-2.21)	.780 (.007)	.788 (.774-.802)
	Q90+	2.18 (1.85-2.57)	.779 (.007)	.787 (.773-.801)
	Q95+	2.33 (1.89-2.87)	.777 (.007)	.785 (.771-.798)
	Q99+	2.35 (1.54-3.59)	.773 (.007)	.781 (.767-.795)
**CHDPRS**		1.78 (1.67-1.91)	.800 (.007)	.808 (.795-.822)
	Q80+	2.39 (2.09-2.74)	.786 (.007)	.794 (.781-.808)
	Q90+	2.51 (2.14-2.95)	.782 (.007)	.790 (.776-.804)
	Q95+	3.14 (2.58-3.82)	.778 (.007)	.787 (.772-.800)
	Q99+	3.52 (2.36-5.26)	.774 (.007)	.783 (.769-.796)
**CHDBioPRS**		1.88 (1.75-2.01)	.806 (.007)	.811 (.798-.824)
	Q80+	2.72 (2.37-3.11)	.791 (.007)	.799 (.786-.812)
	Q90+	2.64 (2.24-3.09)	.784 (.007)	.792 (.778-.805)
	Q95+	3.28 (2.70-3.98)	.783 (.007)	.791 (.777-.804)
	Q99+	4.53 (3.21-6.40)	.776 (.007)	.785 (.771-.798)

Baseline model includes age, sex and the first 10 principal components of genetic structure. Other models add a PRS to the baseline model. For each PRS, the HR is for CHD and per 1 SD of PRS. For each quantile (Q), the HR is for the indicator of the risk group vs. all the others. For example, Q80+ compares the individuals with the highest 20% of PRSes to the remaining 80%. C-Index is from Cox regression model. AUC is computed from a roc (pROC package) call using the prediction results of a CHD logistic regression model adjusted for the baseline parameters.

UKB=UK Biobank, PRS=Polygenic risk score, CHD=Coronary heart disease, HR=Hazard ratio, CI=Confidence interval, SD=Standard deviation, Q=Quantile, SE=Standard error, AUC=Area under the curve, BioPRS=PRS constructed from selected biomarkers, CHDPRS=standard CHD PRS, CHDBioPRS=Risk score integrating BioPRS and CHDPRS

Supplemental Table 5: Results from FinnGen data for quantile levels of PRSes

Model	Quantile	HR for CHD (95% CI)	C-Index (SE)	AUC (95% CI)
**Baseline model**				.724 (.722-.727)
**BioPRS**		1.27 (1.26-1.29)	.696 (.002)	.734 (.732-.737)
	Q80+	1.50 (1.46-1.55)	.688 (.002)	.730 (.727-.732)
	Q90+	1.56 (1.51-1.62)	.685 (.002)	.728 (.725-.731)
	Q95+	1.68 (1.60-1.75)	.683 (.002)	.727 (.724-.730)
	Q99+	1.84 (1.67-2.02)	.681 (.002)	.725 (.722-.728)
**CHDPRS**		1.57 (1.55-1.59)	.721 (.002)	.752 (.750-.755)
	Q80+	2.09 (2.03-2.14)	.703 (.002)	.741 (.738-.743)
	Q90+	2.23 (2.17-2.31)	.696 (.002)	.737 (.734-.739)
	Q95+	2.48 (2.38-2.58)	.691 (.002)	.733 (.730-.736)
	Q99+	3.12 (2.89-3.37)	.683 (.002)	.727 (.724-.730)
**CHDBioPRS**		1.60 (1.58-1.62)	.725 (.002)	.755 (.752-.758)
	Q80+	2.21 (2.1752.27)	.706 (.002)	.743 (.740-.746)
	Q90+	2.32 (2.25-2.40)	.698 (.002)	.738 (.735-.740)
	Q95+	2.52 (2.43-2.62)	.692 (.002)	.733 (.731-.736)
	Q99+	3.24 (2.99-3.50)	.684 (.002)	.727 (.724-.730)

Baseline model includes age, sex and the first 10 principal components of genetic structure. Other models add a PRS to the baseline model. For each PRS, the HR is for CHD and per 1 SD of PRS. For each quantile (Q), the HR is for the indicator of the risk group vs. all the others. For example, Q80+ compares the individuals with the highest 20% of PRSes to the remaining 80%. C-Index is from Cox regression model. AUC is computed from a roc (pROC package) call using the prediction results of a CHD logistic regression model adjusted for the baseline parameters.

UKB=UK Biobank, PRS=Polygenic risk score, CHD=Coronary heart disease, HR=Hazard ratio, CI=Confidence interval, SD=Standard deviation, Q=Quantile, SE=Standard error, AUC=Area under the curve, BioPRS=PRS constructed from selected biomarkers, CHDPRS=standard CHD PRS, CHDBioPRS=Risk score integrating BioPRS and CHDPRS

Supplemental Table 6: Results from FinnGen early CHD onset (age <= 55) data for quantile levels of PRSes

Model	Quantile	HR for CHD (95% CI)	C-Index (SE)	AUC (95% CI)
**Baseline model**				.733 (.727-.739)
**BioPRS**		1.51 (1.47-1.55)	.756 (.003)	.759 (.752-.765)
	Q80+	1.88 (1.77-1.99)	.744 (.003)	.746 (.740-.753)
	Q90+	2.01 (1.87-2.16)	.741 (.003)	.743 (.737-.750)
	Q95+	2.20 (2.02-2.41)	.738 (.003)	.741 (.735-.748)
	Q99+	2.52 (2.11-3.02)	.735 (.003)	.737 (.731-.744)
**CHDPRS**		2.01 (1.95-2.07)	.781 (.003)	.784 (.778-.791)
	Q80+	3.05 (2.88-3.23)	.761 (.003)	.764 (.758-.770)
	Q90+	3.43 (3.22-3.66)	.756 (.003)	.759 (.752-.765)
	Q95+	3.84 (3.56-4.14)	.749 (.003)	.752 (.745-.758)
	Q99+	5.34 (4.71-6.06)	.739 (.003)	.742 (.736-.748)
**CHDBioPRS**		2.10 (2.04-2.16)	.788 (.003)	.791 (.785-.797)
	Q80+	3.42 (3.24-3.62)	.767 (.003)	.770 (.763-.776)
	Q90+	3.60 (3.39-3.84)	.757 (.003)	.760 (.753-.766)
	Q95+	4.04 (3.76-4.35)	.749 (.003)	.752 (.746-.759)
	Q99+	5.02 (4.40-5.72)	.738 (.003)	.741 (.735-.748)

Baseline model includes age, sex and the first 10 principal components of genetic structure. Other models add a PRS to the baseline model. For each PRS, the HR is for CHD and per 1 SD of PRS. For each quantile (Q), the HR is for the indicator of the risk group vs. all the others. For example, Q80+ compares the individuals with the highest 20% of PRSes to the remaining 80%. C-Index is from Cox regression model. AUC is computed from a roc (pROC package) call using the prediction results of a CHD logistic regression model adjusted for the baseline parameters.
Censoring applied for controls at age 55.

UKB=UK Biobank, PRS=Polygenic risk score, CHD=Coronary heart disease, HR=Hazard ratio, CI=Confidence interval, SD=Standard deviation, Q=Quantile, SE=Standard error, AUC=Area under the curve, BioPRS=PRS constructed from selected biomarkers, CHDPRS=standard CHD PRS, CHDBioPRS=Risk score integrating BioPRS and CHDPRS

Supplemental Table 7: Results from UKB Test early CHD onset (age <= 55) data for quantile levels of PRSes

Model	Quantile	HR for CHD(95% CI)	C-Index (SE)	AUC (95% CI)
**Baseline**				.736 (.711-.762)
**BioPRS**		1.60 (1.43-1.78)	.763 (.012)	.765 (.740-.788)
	Q80+	2.34 (1.87-2.94)	.755 (.012)	.757 (.732-.781)
	Q90+	2.81 (2.17-3.63)	.752 (.013)	.754 (.729-.779)
	Q95+	2.89 (2.10-3.99)	.746 (.013)	.748 (.723-.772)
	Q99+	3.31 (1.81-6.04)	.740 (.013)	.741 (.716-.766)
**CHDPRS**		1.90 (1.69-2.13)	.777 (.012)	.780 (.755-.804)
	Q80+	2.83 (2.26-3.56)	.766 (.012)	.768 (.744-.792)
	Q90+	2.91 (2.24-3.78)	.754 (.012)	.756 (.732-.781)
	Q95+	3.56 (2.62-4.86)	.751 (.012)	.753 (.729-.777)
	Q99+	5.51 (3.20-9.49)	.741 (.013)	.743 (.718-.768)
**CHDBioPRS**		2.07 (1.85-2.32)	.788 (.012)	.790 (.766-.814)
	Q80+	3.51 (2.81-4.39)	.776 (.012)	.778 (.755-.801)
	Q90+	3.58 (2.79-4.60)	.762 (.012)	.764 (.740-.789)
	Q95+	4.16 (3.09-5.60)	.755 (.012)	.757 (.732-.782)
	Q99+	5.15 (3.05-8.70)	.742 (.013)	.743 (.719-.768)

Baseline model includes age, sex and the first 10 principal components of genetic structure. Other models add a PRS to the baseline model. For each PRS, the HR is for CHD and per 1 SD of PRS. For each quantile (Q), the HR is for the indicator of the risk group vs. all the others. For example, Q80+ compares the individuals with the highest 20% of PRSes to the remaining 80%. C-Index is from Cox regression model. AUC is computed from a roc (pROC package) call using the prediction results of a CHD logistic regression model adjusted for the baseline parameters.
Censoring applied for controls at age 55.

UKB=UK Biobank, PRS=Polygenic risk score, CHD=Coronary heart disease, HR=Hazard ratio, CI=Confidence interval, SD=Standard deviation, Q=Quantile, SE=Standard error, AUC=Area under the curve, BioPRS=PRS constructed from selected biomarkers, CHDPRS=standard CHD PRS, CHDBioPRS=Risk score integrating BioPRS and CHDPRS

Supplemental Table 8: Results from UKB Test male data for quantile levels of PRSes

Model	Quantile	HR for CHD (95% CI)	C-Index (SE)	AUC (95% CI)
**Baseline model**				.710 (.693-.727)
**BioPRS**		1.42 (1.32-1.53)	.720 (.009)	.733 (.716-.751)
	Q80+	1.85 (1.58-2.16)	.711 (.009)	.725 (.707-.742)
	Q90+	2.12 (1.75-2.56)	.708 (.009)	.722 (.705-.739)
	Q95+	2.13 (1.66-2.74)	.703 (.009)	.717 (.700-.735)
	Q99+	2.52 (1.58-4.03)	.698 (.009)	.712 (.695-.730)
**CHDPRS**		1.72 (1.60-1.86)	.744 (.009)	.757 (.740-.774)
	Q80+	2.54 (2.18-2.97)	.726 (.009)	.739 (.721-.756)
	Q90+	2.94 (2.46-3.51)	.721 (.009)	.734 (.717-.751)
	Q95+	2.79 (2.22-3.52)	.707 (.009)	.721 (.704-.738)
	Q99+	3.80 (2.53-5.72)	.702 (.009)	.716 (.699-.733)
**CHDBioPRS**		1.84 (1.70-1.98)	.752 (.009)	.765 (.748-.782)
	Q80+	2.80 (2.41-3.26)	.733 (.009)	.746 (.729-.764)
	Q90+	3.03 (2.54-3.61)	.721 (.009)	.734 (.717-.752)
	Q95+	3.23 (2.59-4.02)	.714 (.009)	.728 (.710-.745)
	Q99+	4.11 (2.77-6.08)	.703 (.009)	.717 (.699-.734)

Baseline model includes age, sex and the first 10 principal components of genetic structure. Other models add a PRS to the baseline model. For each PRS, the HR is for CHD and per 1 SD of PRS. For each quantile (Q), the HR is for the indicator of the risk group vs. all the others. For example, Q80+ compares the individuals with the highest 20% of PRSes to the remaining 80%. C-Index is from Cox regression model. AUC is computed from a roc (pROC package) call using the prediction results of a CHD logistic regression model adjusted for the baseline parameters.

UKB=UK Biobank, PRS=Polygenic risk score, CHD=Coronary heart disease, HR=Hazard ratio, CI=Confidence interval, SD=Standard deviation, Q=Quantile, SE=Standard error, AUC=Area under the curve, BioPRS=PRS constructed from selected biomarkers, CHDPRS=standard CHD PRS, CHDBioPRS=Risk score integrating BioPRS and CHDPRS

Supplemental Table 9: Results from UKB Test female data for quantile levels of PRSes

Model	Quantile	HR for CHD (95% CI)	C-Index (SE)	AUC (95% CI)
**Baseline model**				.571 (.567-.575)
**BioPRS**		1.27 (1.25-1.29)	.606 (.002)	.612 (.608-.616)
	Q80+	1.48 (1.44-1.53)	.593 (.002)	.599 (.595-.603)
	Q90+	1.56 (1.50-1.63)	.588 (.002)	.596 (.592-.600)
	Q95+	1.68 (1.59-1.77)	.584 (.002)	.593 (.589-.597)
	Q99+	1.75 (1.56-1.96)	.578 (.002)	.586 (.582-.590)
**CHDPRS**		1.58 (1.55-1.60)	.655 (.002)	.652 (.648-.655)
	Q80+	2.09 (2.03-2.16)	.624 (.002)	.625 (.621-.629)
	Q90+	2.22 (2.14-2.31)	.608 (.002)	.614 (.610-.618)
	Q95+	2.45 (2.33-2.57)	.596 (.002)	.603 (.599-.607)
	Q99+	3.10 (2.83-3.40)	.581 (.002)	.590 (.586-.594)
**CHDBioPRS**		1.61 (1.59-1.63)	.663 (.002)	.657 (.653-.661)
	Q80+	2.21 (2.15-2.28)	.629 (.002)	.631 (.627-.635)
	Q90+	2.33 (2.24-2.41)	.611 (.002)	.616 (.612-.620)
	Q95+	2.51 (2.40-2.64)	.598 (.002)	.604 (.600-.608)
	Q99+	2.98 (2.71-3.27)	.581 (.002)	.589 (.585-.593)

Baseline model includes age, sex and the first 10 principal components of genetic structure. Other models add a PRS to the baseline model. For each PRS, the HR is for CHD and per 1 SD of PRS. For each quantile (Q), the HR is for the indicator of the risk group vs. all the others. For example, Q80+ compares the individuals with the highest 20% of PRSes to the remaining 80%. C-Index is from Cox regression model. AUC is computed from a roc (pROC package) call using the prediction results of a CHD logistic regression model adjusted for the baseline parameters.

UKB=UK Biobank, PRS=Polygenic risk score, CHD=Coronary heart disease, HR=Hazard ratio, CI=Confidence interval, SD=Standard deviation, Q=Quantile, SE=Standard error, AUC=Area under the curve, BioPRS=PRS constructed from selected biomarkers, CHDPRS=standard CHD PRS, CHDBioPRS=Risk score integrating BioPRS and CHDPRS

Supplemental Table 10: Results from FinnGen female data for quantile levels of PRSes

Model	Quantile	HR for CHD (95% CI)	C-Index (SE)	AUC (95% CI)
**Baseline model**				.710 (.705-.710)
**BioPRS**		1.27 (1.24-1.30)	.625 (.004)	.720 (.715-.725)
	Q80+	1.50 (1.42-1.57)	.611 (.004)	.715 (.710-.721)
	Q90+	1.59 (1.49-1.69)	.607 (.004)	.714 (.709-.719)
	Q95+	1.67 (1.53-1.82)	.604 (.004)	.713 (.708-.718)
	Q99+	1.88 (1.57-2.24)	.598 (.004)	.711 (.706-.716)
**CHDPRS**		1.53 (1.50-1.57)	.657 (.004)	.738 (.733-.743)
	Q80+	2.05 (1.95-2.15)	.638 (.004)	.728 (.723-.733)
	Q90+	2.24 (2.12-2.38)	.629 (.004)	.725 (.720-.730)
	Q95+	2.51 (2.34-2.70)	.619 (.004)	.722 (.717-.727)
	Q99+	3.20 (2.80-3.66)	.604 (.004)	.715 (.710-.720)
**CHDBioPRS**		1.56 (1.53-1.60)	.665 (.005)	.741 (.736-.746)
	Q80+	2.14 (2.03-2.24)	.645 (.004)	.730 (.725-.735)
	Q90+	2.29 (2.16-2.42)	.631 (.004)	.726 (.721-.731)
	Q95+	2.50 (2.32-2.69)	.621 (.004)	.722 (.717-.727)
	Q99+	3.70 (3.23-4.23)	.605 (.004)	.715 (.710-.720)

Baseline model includes age and the first 10 principal components of genetic structure. Other models add a PRS to the baseline model. For each PRS, the HR is for CHD and per 1 SD of PRS. For each quantile (Q), the HR is for the indicator of the risk group vs. all the others. For example, Q80+ compares the individuals with the highest 20% of PRSes to the remaining 80%. C-Index is from Cox regression model. AUC is computed from a roc (pROC package) call using the prediction results of a CHD logistic regression model adjusted for the baseline parameters.

UKB=UK Biobank, PRS=Polygenic risk score, CHD=Coronary heart disease, HR=Hazard ratio, CI=Confidence interval, SD=Standard deviation, Q=Quantile, SE=Standard error, AUC=Area under the curve, BioPRS=PRS constructed from selected biomarkers, CHDPRS=standard CHD PRS, CHDBioPRS=Risk score integrating BioPRS and CHDPRS

Supplemental Table 11: Results from UKB Test female data for quantile levels of PRSes

Model	Quantile	HR for CHD (95% CI)	C-Index (SE)	AUC (95% CI)
**Baseline model**				.726 (.694-.757)
**BioPRS**		1.53 (1.34-1.75)	.746 (.015)	.750 (.719-.780)
	Q80+	1.75 (1.31-2.35)	.731 (.016)	.736 (.705-.767)
	Q90+	2.11 (1.48-2.99)	.731 (.016)	.735 (.703-.766)
	Q95+	2.43 (1.56-3.78)	.730 (.016)	.734 (.703-.765)
	Q99+	2.72 (1.12-6.63)	.722 (.016)	.727 (.695-.758)
**CHDPRS**		1.72 (1.50-1.98)	.756 (.015)	.760 (.730-.790)
	Q80+	2.16 (1.62-2.88)	.735 (.016)	.739 (.708-.770)
	Q90+	2.40 (1.71-3.37)	.731 (.016)	.736 (.704-.767)
	Q95+	3.86 (2.66-5.59)	.737 (.016)	.741 (.709-.772)
	Q99+	3.81 (1.86-7.80)	.728 (.016)	.732 (.701-.763)
**CHDBioPRS**		1.86 (1.62-2.13)	.766 (.015)	.770 (.741-.799)
	Q80+	2.78 (2.11-3.66)	.756 (.015)	.756 (.726-.786)
	Q90+	2.72 (1.96-3.79)	.736 (.016)	.741 (.709-.772)
	Q95+	4.03 (2.77-5.85)	.739 (.016)	.743 (.712-.775)
	Q99+	5.15 (2.77-9.58)	.726 (.016)	.730 (.698-.761)

Baseline model includes age, sex and the first 10 principal components of genetic structure. Other models add a PRS to the baseline model. For each PRS, the HR is for CHD and per 1 SD of PRS. For each quantile (Q), the HR is for the indicator of the risk group vs. all the others. For example, Q80+ compares the individuals with the highest 20% of PRSes to the remaining 80%. C-Index is from Cox regression model. AUC is computed from a roc (pROC package) call using the prediction results of a CHD logistic regression model adjusted for the baseline parameters.

UKB=UK Biobank, PRS=Polygenic risk score, CHD=Coronary heart disease, HR=Hazard ratio, CI=Confidence interval, SD=Standard deviation, Q=Quantile, SE=Standard error, AUC=Area under the curve, BioPRS=PRS constructed from selected biomarkers, CHDPRS=standard CHD PRS, CHDBioPRS=Risk score integrating BioPRS and CHDPRS

Supplemental Table 12: Results from FinnGen female early onset (age <= 60) data for quantile levels of PRSes

Model	Quantile	HR for CHD (95% CI)	C-Index (SE)	AUC (95% CI)
**Baseline model**				.636 (.625-.646)
**BioPRS**		1.44 (1.38-1.51)	.668 (.006)	.670 (.659-.681)
	Q80+	1.82 (1.65-2.00)	.655 (.006)	.657 (.651-.668)
	Q90+	1.93 (1.72-2.17)	.650 (.006)	.651 (.640-.663)
	Q95+	2.03 (1.74-2.36)	.645 (.006)	.646 (.635-.658)
	Q99+	2.08 (1.51-2.86)	.639 (.006)	.640 (.629-.652)
**CHDPRS**		1.82 (1.74-1.90)	.697 (.006)	.698 (.686-.710)
	Q80+	2.64 (2.41-2.91)	.676 (.006)	.677 (.666-.689)
	Q90+	2.90 (2.61-3.23)	.668 (.006)	.670 (.658-.681)
	Q95+	3.40 (3.00-3.85)	.659 (.006)	.661 (.649-.672)
	Q99+	4.90 (3.99-6.02)	.647 (.006)	.649 (.637-.660)
**CHDBioPRS**		1.90 (1.81-1.99)	.707 (.006)	.709 (.697-.720)
	Q80+	2.97 (2.71-3.26)	.684 (.006)	.686 (.674-.698)
	Q90+	3.06 (2.75-3.39)	.671 (.006)	.673 (.661-.684)
	Q95+	3.39 (3.00-3.84)	.662 (.006)	.663 (.652-.675)
	Q99+	4.80 (3.88-5.92)	.646 (.006)	.647 (.636-.658)

Baseline model includes age and the first 10 principal components of genetic structure. Other models add a PRS to the baseline model. For each PRS, the HR is for CHD and per 1 SD of PRS. For each quantile (Q), the HR is for the indicator of the risk group vs. all the others. For example, Q80+ compares the individuals with the highest 20% of PRSes to the remaining 80%. C-Index is from Cox regression model. AUC is computed from a roc (pROC package) call using the prediction results of a CHD logistic regression model adjusted for the baseline parameters.
Censoring applied for controls at age 60.

UKB=UK Biobank, PRS=Polygenic risk score, CHD=Coronary heart disease, HR=Hazard ratio, CI=Confidence interval, SD=Standard deviation, Q=Quantile, SE=Standard error, AUC=Area under the curve, BioPRS=PRS constructed from selected biomarkers, CHDPRS=standard CHD PRS, CHDBioPRS=Risk score integrating BioPRS and CHDPRS

Supplemental Table 13: Results from FinnGen male early onset (age <= 50) data for quantile levels of PRSes

Model	Quantile	HR for CHD (95% CI)	C-Index (SE)	AUC (95% CI)
**Baseline model**				.578 (.567-.590)
**BioPRS**		1.53 (1.47-1.60)	.639 (.006)	.641 (.629-.653)
	Q80+	2.02 (1.85-2.22)	.611 (.006)	.613 (.600-.625)
	Q90+	2.09 (1.87-2.33)	.597 (.006)	.598 (.586-.611)
	Q95+	2.31 (2.01-2.66)	.593 (.006)	.594 (.582-.607)
	Q99+	2.44 (1.85-3.24)	.584 (.006)	.585 (.572-.597)
**CHDPRS**		2.17 (2.07-2.27)	.706 (.006)	.708 (.697-.720)
	Q80+	3.36 (3.07-3.68)	.657 (.006)	.658 (.646-.671)
	Q90+	3.98 (3.61-4.39)	.642 (.006)	.644 (.631-.656)
	Q95+	4.66 (4.16-5.20)	.624 (.006)	.626 (.613-.638)
	Q99+	5.88 (4.88-7.09)	.593 (.006)	.594 (.582-.607)
**CHDBioPRS**		2.26 (2.16-2.36)	.716 (.006)	.719 (.707-.730)
	Q80+	3.80 (3.47-4.15)	.670 (.006)	.672 (.659-.684)
	Q90+	4.07 (3.70-4.48)	.642 (.006)	.644 (.631-.657)
	Q95+	4.76 (4.26-5.32)	.625 (.006)	.626 (.614-.639)
	Q99+	5.63 (4.66-6.82)	.591 (.006)	.592 (.580-.605)

Baseline model includes age and the first 10 principal components of genetic structure. Other models add a PRS to the baseline model. For each PRS, the HR is for CHD and per 1 SD of PRS. For each quantile (Q), the HR is for the indicator of the risk group vs. all the others. For example, Q80+ compares the individuals with the highest 20% of PRSes to the remaining 80%. C-Index is from Cox regression model. AUC is computed from a roc (pROC package) call using the prediction results of a CHD logistic regression model adjusted for the baseline parameters.
Censoring applied for controls at age 50.

UKB=UK Biobank, PRS=Polygenic risk score, CHD=Coronary heart disease, HR=Hazard ratio, CI=Confidence interval, SD=Standard deviation, Q=Quantile, SE=Standard error, AUC=Area under the curve, BioPRS=PRS constructed from selected biomarkers, CHDPRS=standard CHD PRS, CHDBioPRS=Risk score integrating BioPRS and CHDPRS

Supplemental Table 14: Results from UKB Test early onset (CHD age <= 60) female data for quantile levels of PRSes

**Model**	**Quantile**	**HR for CHD (95% CI)**	**C-Index (SE)**	**AUC (95% CI)**
**Baseline model**				.654 (.609-.699)
**BioPRS**		1.57 (1.31-1.89)	.701 (.022)	.702 (.659-.746)
	Q80+	1.90 (1.29-2.79)	.676 (.023)	.677 (.631-.723)
	Q90+	2.19 (1.39-3.45)	.668 (.023)	.669 (.622-.715)
	Q95+	2.36 (1.33-4.21)	.662 (.023)	.663 (.619-.707)
	Q99+	1.62 (0.40-6.57)	.655 (.023)	.656 (.611-.702)
**CHDPRS**		1.72 (1.52-2.15)	.715 (.021)	.717 (.674-.759)
	Q80+	2.07 (1.41-3.04)	.681 (.023)	.682 (.636-.728)
	Q90+	2.48 (1.59-3.86)	.677 (.024)	.678 (.631-.724)
	Q95+	3.81 (2.33-6.25)	.681 (.023)	.682 (.636-.728)
	Q99+	4.30 (1.72-10.74)	.666 (.022)	.667 (.624-.711)
**CHDBioPRS**		1.89 (1.57-2.27)	.732 (.021)	.733 (.691-.775)
	Q80+	3.18 (2.21-4.59)	.715 (.023)	.715 (.671-.760)
	Q90+	2.65 (1.71-4.10)	.681 (.023)	.681 (.635-.728)
	Q95+	3.92 (2.40-6.40)	.683 (.023)	.684 (.638-.730)
	Q99+	4.03 (1.61-10.13)	.663 (.023)	.664 (.619-.709)

Baseline model includes age and the first 10 principal components of genetic structure. Other models add a PRS to the baseline model. For each PRS, the HR is for CHD and per 1 SD of PRS. For each quantile (Q), the HR is for the indicator of the risk group vs. all the others. For example, Q80+ compares the individuals with the highest 20% of PRSes to the remaining 80%. C-Index is from Cox regression model. AUC is computed from a roc (pROC package) call using the prediction results of a CHD logistic regression model adjusted for the baseline parameters.
Censoring applied for controls at age 60.

UKB=UK Biobank, PRS=Polygenic risk score, CHD=Coronary heart disease, HR=Hazard ratio, CI=Confidence interval, SD=Standard deviation, Q=Quantile, SE=Standard error, AUC=Area under the curve, BioPRS=PRS constructed from selected biomarkers, CHDPRS=standard CHD PRS, CHDBioPRS=Risk score integrating BioPRS and CHDPRS

Supplemental Table 15: Results from UKB Test early onset (CHD age <= 50) male data for quantile levels of PRSes

Model	Quantile	HR for CHD(95% CI)	C-Index (SE)	AUC (95% CI)
**Baseline model**				**.612 (.563-.660)**
**BioPRS**		1.36 (1.15-1.62)	.636 (.025)	.637 (.588-.686)
	Q80+	1.66 (1.13-2.44)	.629 (.024)	.630 (.582-.677)
	Q90+	2.70 (1.78-4.09)	.645 (.025)	.645 (.596-.695)
	Q95+	2.53 (1.48-4.35)	.625 (.025)	.626 (.577-.675)
	Q99+	4.82 (2.12-10.94)	.621 (.025)	.622 (.572-.672)
**CHDPRS**		1.81 (1.52-2.15)	.675 (.025)	.676 (.627-.725)
	Q80+	2.75 (1.91-3.92)	.665 (.024)	.667 (.618-.715)
	Q90+	3.40 (2.28-5.05)	.657 (.025)	.657 (.606-.707)
	Q95+	4.04 (2.54-6.42)	.637 (.026)	.638 (.587-.689)
	Q99+	9.00 (4.70-17.21)	.629 (.025)	.630 (.579-.680)
**CHDBioPRS**		1.87 (1.56-2.23)	.687 (.025)	.688 (.639-.737)
	Q80+	2.73 (1.91-3.92)	.670 (.024)	.671 (.624-.718)
	Q90+	3.66 (2.48-5.41)	.673 (.025)	.674 (.625-.723)
	Q95+	5.10 (3.30-7.88)	.664 (.026)	.664 (.614-.715)
	Q99+	7.94 (4.02-15.68)	.633 (.006)	.634 (.583-.684)

Baseline model includes age and the first 10 principal components of genetic structure. Other models add a PRS to the baseline model. For each PRS, the HR is for CHD and per 1 SD of PRS. For each quantile (Q), the HR is for the indicator of the risk group vs. all the others. For example, Q80+ compares the individuals with the highest 20% of PRSes to the remaining 80%. C-Index is from Cox regression model. AUC is computed from a roc (pROC package) call using the prediction results of a CHD logistic regression model adjusted for the baseline parameters.
Censoring applied for controls at age 60.

PRS=Polygenic risk score, CHD=Coronary heart disease, HR=Hazard ratio, CI=Confidence interval, SD=Standard deviation, Quant(Q)=Quantile, SE=Standard error, AUC=Area under the curve, BioPRS=PRS constructed from selected biomarkers, CHDPRS=standard CHD PRS, CHDBioPRS=Risk score integrated from BioPRS and CHDPRS

Supplemental Table 16: Net reclassification improvement (NRI) score comparison between CHDPRS (standard) and BioPRS (new)*

NRI model comparisons	NRI	NRI+	NRI-
**UKB Training data**			
CHDPRS (std) CHDBioPRS (new)	0.224	0.119	0.105
CHDPRS-Score2 (std) CHDBioPRS-Score2 (new)	0.201	0.094	0.107
Female CHDPRS (std) CHDBioPRS (new)	0.263	0.137	0.126
Female CHDPRS-Score2 (std) CHDBioPRS-Score2 (new)	0.201	0.096	0.104
Male CHDPRS (std) CHDBioPRS (new)	0.202	0.105	0.097
Male CHDPRS-Score2 (std) CHDBioPRS-Score2 (new)	0.183	0.089	0.094
Early onset CHDPRS (std) CHDBioPRS (new)	0.274	0.145	0.129
Early onset CHDPRS-Score2 (std) CHDBioPRS-Score2 (new)	0.221	0.100	0.121
**UKB Test data**			
CHDPRS (std) CHDBioPRS (new)	0.239	0.131	0.108
CHDPRS-Score2 (std) CHDBioPRS-Score2 (new)	0.252	0.121	0.131
Early onset CHDPRS (std) CHDBioPRS (new)	0.301	0.157	0.144
Early onset CHDPRS-Score2 (std) CHDBioPRS-Score2 (new)	0.326	0.169	0.157
**FinnGen**			
CHDPRS (std) CHDBioPRS (new)	0.102	0.057	0.045
Female CHDPRS (std) CHDBioPRS (new)	0.104	0.078	0.026
Male CHDPRS (std) CHDBioPRS (new)	0.088	0.037	0.051
Early onset CHDPRS (std) CHDBioPRS (new)	0.139	0.066	0.073

NRI is a sum of two measures (NRI+ and NRI-). NRI+ is the difference between the proportions of cases who were (correctly) assigned to a higher risk by the new classification than by the standard one, and those who were (wrongly) reassigned to have a lower risk. NRI- is a difference between proportions of controls who were (correctly) reassigned to a lower risk and those who were (wrongly) reassigned to a higher risk. Positive values of NRI show that the new classification improves over the standard one.

*'Diff standard mode' in nricens function of nricens R-package was applied and 'new' and 'std' refer to the arguments of the function. The analyses were adjusted for age, sex (when relevant) and the first ten principal components of genetic structure.

UKB=UK Biobank, PRS=Polygenic risk score, CHD=Coronary heart disease, BioPRS=PRS constructed from selected biomarkers, CHDPRS=standard CHD PRS, CHDBioPRS=Risk score integrated from BioPRS and CHDPRS

Supplemental Table 17: Coefficients of biomarker PRSes from a joint Cox regression model of CHD

Biomarker	Coefficient
**(A) Full sample**	
Apo-A1	-0.037
ApoB	0.04
CPD	0.152
CREA	0.003
C-Reactive Protein	0.03
HbA1c	0.046
HDL	-0.05
LDL	0.102
SBP	0.194
TRIG	0.089
**(B) Female only**	
ApoB	0.002
CPD	0.209
C-Reactive Protein	0.034
HbA1c	0.035
HDL	-0.129
LDL	0.142
SBP	0.261
TRIG	0.021
**(C) Male only**	
ApoB	0.055
CPD	0.124
C-Reactive Protein	0.028
HbA1c	0.054
HDL	-0.069
LDL	0.095
SBP	0.168
TRIG	0.101

Coefficients are from a joint Cox regression model using incident CHD as the outcome in the UKB Training data and adjusted for age and the first ten principal components of genetic population structure.

CHD=Coronary heart disease, PRS=Polygenic risk score UKB=UK Biobank, CPD=Cigarettes per day, CREA=creatinine, HbA1c=Hemoglobin-A1c, HDL=High-density lipoprotein, LDL=Low-density lipoprotein, SBP=Systolic blood pressure, TRIG=Triglyceride

Supplemental Table 18: Likelihood ratio test (LRT) statistic of Cox models having different scores as predictors

	BioPRS	CHDPRS	CHDBioPRS	Score2	CHDPRS_Score2	CHDBioPRS_Score2
UKB Training	10689	11890	12693	10219	12772	13211
UKB Training females	1653	1806	2036	1754	2234	2312
UKB Training males	4164	5241	5789	3739	5773	6095
UKB Validation	4644	5182	5487	4630	5681	5822
UKB Test	1160	1296	1371	1131	1392	1446
UKB Test females	201	220.5	243	204	253	264
UKB Test males	497	610.8	667	474	684	722
UKB Test early onset	296	336,7	373	286	385	409
FinnGen	9825	13172	13678	na	na	na
FinnGen females	890	1721	1871	na	na	na
FinnGen males	2136	4627	4990	na	na	na
FinnGen early onset	4573	5969	6294	na	na	na

CHD=Coronary heart disease, PRS=Polygenic risk score, UKB=UK Biobank, BioPRS=PRS constructed from selected biomarkers, CHDPRS=standard CHD PRS, CHDBioPRS=Risk score integrated from BioPRS and CHDPRS, SCORE2=calculated from subject data on age, total cholesterol, high-density lipoprotein, systolic blood pressure, diabetes event and smoking status

Supplemental Table 19: Results of SCORE2 and integration of SCORE2 with PRSes. Hazard ratios (HR) are per 1 SD of SCORE2 (or the combined predictor)

**Cohort**	**SCORE2 HR (95% CI)**	**C-INDEX (SE)**	**SCORE2 +CHDPRS HR (95% CI)**	**C-INDEX (SE)**	**SCORE2 + CHDBioPRS HR (95% CI)**	**C-INDEX (SE)**
UKB Training	1.33 (1.31-1.35)	0.762 (0.002)	1.72 (1.69-1.75)	0.787 (0.002)	1.78 (1.75-1.81)	0.791 (0.002)
UKB Validation	1.37 (1.34-1.40)	0.765 (0.003)	1.75 (1.70-1.80)	0.789 (0.003)	1.80 (1.75-1.84)	0.792 (0.003)
UKB Test	1.34 (1.28-1.41)	0.781 (0.006)	1.80 (1.70-1.90)	0.805 (0.006)	1.89 (1.78-2.00)	0.809 (0.006)
UKB Test females	1.41 (1.30-1.53)	0.750 (0.014)	1.67 (1.53-1.83)	0.774 (0.014)	1.73 (1.58-1.89)	0.779 (0.014)
UKB Test males	1.34 (1.27-1.42)	0.713 (0.008)	1.72 (1.68-1.75)	0.751 (0.008)	1.78 (1.74-1.81)	0.757 (0.008)
UKB Test early onset	1.42 (1.32-1.54)	0.758 (0.013)	1.95 (1.77-2.16)	0.792 (0.012)	2.08 (1.88-2.30)	0.797 (0.012)

Results are from Cox model adjusted for age, sex (when relevant) and the first 10 principal components of genetic population structure.
The hazard ratios are for CHD and per 1 standard deviation of the predictor.
CHD=Coronary heart disease, PRS=Polygenic risk score, HR=Hazard ratio, CI=confidence interval, SE=Standard error, UKB=UK Biobank, BioPRS=PRS constructed from selected biomarkers, CHDPRS=standard CHD PRS, CHDBioPRS=Risk score integrating BioPRS and CHDPRS, SCORE2=calculated from individual-level data on age, total cholesterol, high-density lipoprotein cholesterol, systolic blood pressure, diabetes status and smoking status.
